# Adoption and implementation of a web-based self-management application “Oncokompas” in routine cancer care: a national pilot study

**DOI:** 10.1007/s00520-018-4591-5

**Published:** 2018-12-18

**Authors:** L. Matthijs de Wit, Cornelia F. van Uden-Kraan, Birgit I. Lissenberg-Witte, Heleen C. Melissant, Margot A.H. Fleuren, Pim Cuijpers, Irma M. Verdonck-de Leeuw

**Affiliations:** 1 Department of Clinical, Neuro- and Developmental Psychology, Amsterdam Public Health research institute, Vrije Universiteit Amsterdam, Amsterdam, The Netherlands; 2 Cancer Center Amsterdam (CCA), Amsterdam, The Netherlands; 3 Amsterdam Public Health research institute, Amsterdam, The Netherlands; 4 Department of Epidemiology and Biostatistics, Amsterdam UMC, Vrije Universiteit Amsterdam, Amsterdam, The Netherlands; 5 Department of Otolaryngology-Head and Neck Surgery, Amsterdam UMC, Vrije Universiteit Amsterdam, 7057, 1007 MB Amsterdam, The Netherlands

**Keywords:** eHealth, Cancer survivors, Adoption, Implementation, Health-related quality of life, Patient-reported outcome measures

## Abstract

**Purpose:**

A web-based self-management application “Oncokompas” was developed to monitor health-related quality of life and to support cancer survivors in finding and obtaining optimal supportive care. Access to this application is provided via a healthcare professional (HCP). The aim of this study was to explore the adoption and implementation of Oncokompas in routine clinical practice and to obtain insights in potentially relevant determinants of implementation.

**Methods:**

A pilot study was carried out among 65 hospitals throughout The Netherlands. HCPs filled out a questionnaire on the implementation of Oncokompas in their organization, consisting of study specific items and items based on the Measurement Instrument for Determinants of Innovations (MIDI). The MIDI comprises 29 determinants in four domains that predict the use of innovations: the innovation itself (Oncokompas), the user (HCP), the organization (hospital), and socio-political context.

**Results:**

In total, 20/65 eligible hospitals agreed to implement Oncokompas (adoption rate 31%). In these 20 adopting hospitals, the majority of the responding HCPs (72/205) in this study (44/61) indicated their patients were offered access to Oncokompas (implementation rate 72%). Comparing those HCPs who did and did not implement Oncokompas, the groups differed significantly on innovation-related (procedural clarity, complexity) and user-related determinants (importance of outcome expectations, professional obligation, social support, and self-efficacy).

**Conclusions:**

During this 1-year study, nationwide adoption rate of Oncokompas was at 31%, and subsequent implementation rate was at 72%. The results of this study contribute to further optimize interventions and strategies to adopt and implement (online) self-management applications in cancer care.

**Electronic supplementary material:**

The online version of this article (10.1007/s00520-018-4591-5) contains supplementary material, which is available to authorized users.

## Introduction

Cancer and the treatment of cancer often have a negative impact on a cancer survivors’ health-related quality of life (HRQOL). Addressing this requires a multidisciplinary and multichannel approach using different strategies. One of these strategies is self-management, and even though its benefits have been recognized [1–3], integration in routine cancer care is still in its early stages. There is an important role for healthcare professionals (HCPs) in informing and encouraging self-management in patients, as patients consider their HCP an important source of information [4]. Self-management support has been defined as “the systematic provision of education and supportive interventions by healthcare staff to increase patients’ skills and confidence in managing their health problems, including regular assessment of progress and problems, goal setting, and problem-solving support" [5]. HCPs have many options and strategies to choose from when it comes to providing self-management support. An example is informing patients of (web-based) tools that facilitate or enable self-management behaviors of cancer survivors [6–8].

The web-based self-management application Oncokompas has been designed to facilitate access to supportive cancer care services. Users measure their HRQOL by means of patient-reported outcome measures (PROMs) targeting over 80 different cancer related HRQOL topics, which are processed by the algorithms built into Oncokompas. All algorithm calculations are based on available cut-off scores, or they are defined based on Dutch practice guidelines, literature searches, and/or consensus by teams of experts (consisting of patients, physicians, nurses, researchers, psychologists, and other experts) [6]. This results in red/orange/green scores on the various topics. These scores are accompanied by automatically generated feedback, information, insights, and tips to deal with problem areas, all tailored to the individual patient. Finally, Oncokompas provides options for supportive cancer care for each HRQOL topic. These options range from (online) self-help options (in case of an orange score) to traditional face-to-face care options, such as a psychologist (in case of a red score), accompanied with contact information and map to the nearest psychologist specialized in cancer extracted from the Dutch Cancer Referral Guide [9], based on the user’s postal code.

Participatory design principles were followed to ensure sustainable usage of Oncokompas, meaning that all stakeholders, including cancer survivors, HCPs, healthcare insurance companies, and researchers were involved in each step of the development process [6, 10–12]. This approach resulted in an eHealth application that fits the needs of cancer survivors and HCPs and is proven to be feasible for usage in clinical practice, combined with good satisfaction rates among cancer survivors' [6, 13]. Oncokompas is designed as a self-help application, based on a feasibility study among HCPs and patients valuing independent use. A supported self-management approach was also explored (blended care), but the importance of empowering the user and respecting their privacy resulted in implementing Oncokompas as a stand-alone self-management tool. Additionally, a supported approach would have led to increased complexity regarding existing working procedures, potentially leading to low uptake. [6, 11, 14]. To stimulate HCP involvement, access to the application occurs via HCPs.

**Fig. 1:**
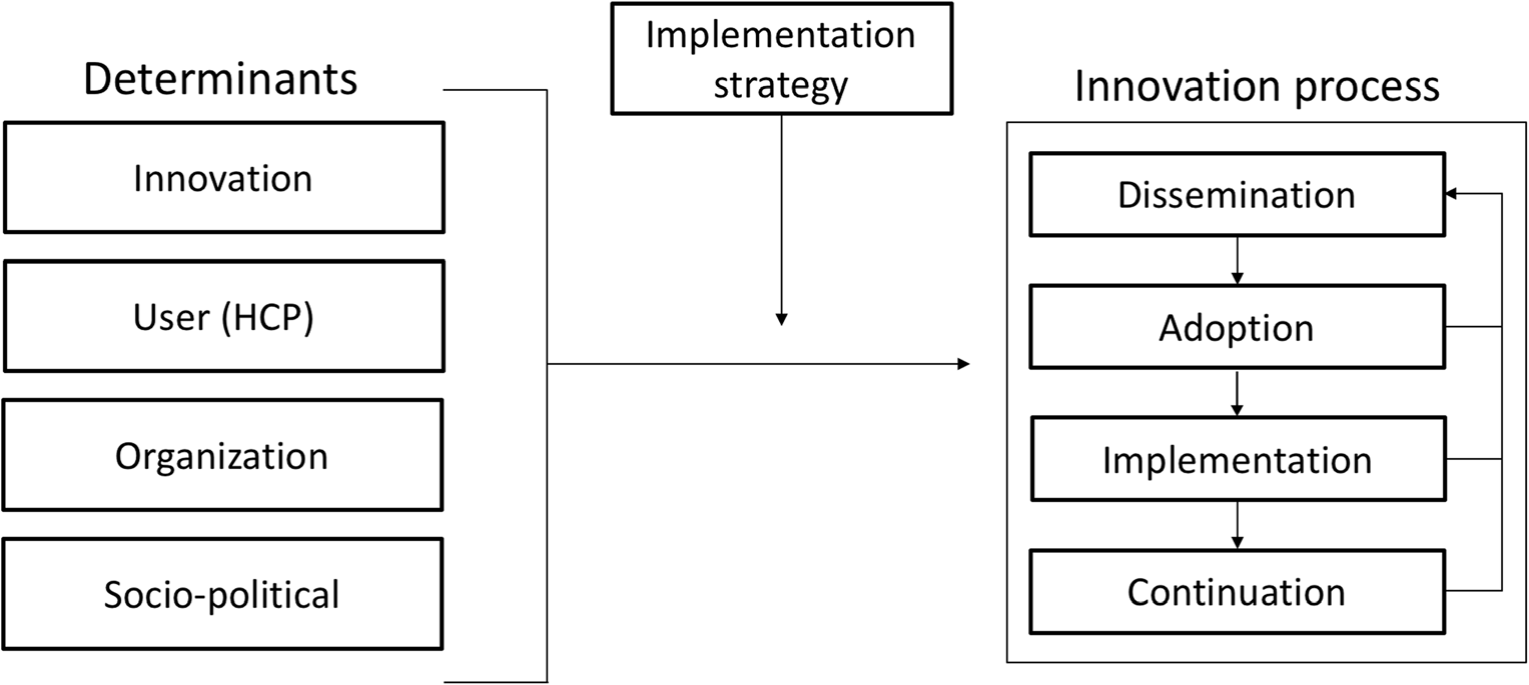
The Measurement Instrument for Determinants of Innovations (MIDI) [20]

The next step was to implement Oncokompas in Dutch oncology settings. Earlier research has shown that successful implementation of innovations, especially e-health applications in healthcare, is difficult to achieve, and many determinants can be of influence [15–18]. In order to facilitate the adoption and implementation of Oncokompas in oncology settings, a comprehensive multifaceted implementation strategy was designed, in which we incorporated insights from earlier studies [6, 19] as well as the requirements for implementation of Oncokompas indicated by HCPs, such as designing Oncokompas in a way that enables independent use by patients [11]. Figure 1 shows a generic framework that has been used for the introduction and evaluation of innovations in healthcare [20]. Each of the four main stages in innovation processes can be thought of as critical phase where the desired change may or may not occur. The transition from one stage to the next can be affected, positively or negatively, by various determinants. A detailed understanding of determinants helps to design an innovation strategy that can achieve real change.

The aim of this study was to investigate the adoption and implementation of Oncokompas in clinical practice and to obtain insights in possible determinants of implementation. Study results are relevant to guide and increase future implementation of (online) self-management interventions in oncology settings.

## Materials and methods

### Design and study setting

In this cross-sectional multicentre pilot study, the adoption and implementation of Oncokompas in routine cancer care were investigated. Adoption was defined as whether a hospital agreed to offer Oncokompas to their patients. Implementation was defined as whether Oncokompas was actually offered to patients of the participating HCPs. At the time of the study (2015–2016), there were 78 hospitals in the Netherlands, who were all informed about Oncokompas by a health insurance company. In total, 13 hospitals were excluded for this study because they were involved in research on the development and effectiveness of Oncokompas [13, 21]. Therefore, the study population in the present study was HCPs in 65 hospitals. In these 65 hospitals, the adoption rate and reasons for not adopting were examined. Second, in the adopting hospitals, implementation of Oncokompas was investigated from the perspective of HCPs involved with implementation of Oncokompas in their hospital.

### Intervention “Oncokompas”

The web-based self-management application “Oncokompas” provides personalized feedback, information, advice, and options for supportive cancer care, based on the situation of the cancer survivor. Oncokompas consists of three components: “measure,” “learn,” and “act” (see Multimedia Appendix A for screenshots of Oncokompas).

1. *Measure* comprises of assessment of PROMs targeting the following quality-of-life domains: physical, psychological and social functioning, healthy lifestyle, and existential issues.
2. *Learn* allows the user to obtain personalized information and advice based on the PROM data provided by the user in the *measure* component.
3. *Act* gives users an overview of options for supportive cancer care services.

Oncokompas is designed to be used before treatment (after diagnosis), during treatment, and in follow-up care by patients who are (going to be) treated with curative intent (cancer survivors). Oncokompas was developed to be used by cancer survivors independently from their HCP, while allowing users to optionally share their Oncokompas results and progress over time with their HCP by bringing a (digital) copy of their Oncokompas dossier.

Implementation of Oncokompas consists for HCPs of the following steps: (1) informing the patient about Oncokompas, (2) logging into Oncokompas with an HCP account, (3) submitting an online form with personal information of the patient: name, e-mail address, date of birth, treatment phase (before/during/after treatment), and home address. Oncokompas sends a personal activation link to the e-mail address of the cancer survivor, who then completes the registration and starts the Measure component as described above.

Oncokompas is considered to be a medical device and is in compliance with Dutch and European laws and regulations (Medical Device Directive [22]). All data are stored safely and encrypted by an enterprise grade hosting company, which is NEN7510 certified (Dutch norm for information security in healthcare).

### Implementation strategy

The multifaceted implementation strategy used for implementing Oncokompas consisted of several discrete implementation strategies [23] and was selected based on consensus among a team of health care providers and researchers. The core elements are listed in Table 1.

**Table 1:** Multifaceted implementation strategy for implementing Oncokompas

1	Readiness and possible barriers and facilitators were identified in a previous feasibility study and assessment of HCP perspectives [6, 11].
2	The Oncokompas team presented Oncokompas to the HCPs involved in oncology care (often centralized in an oncological committee, which is an executive medical board), highlighting the participatory design approach, features of Oncokompas, benefits for patients and HCPs, and ease of use of the application. In case a hospital adopted Oncokompas, an instructional meeting was organized for staff that was going to work with Oncokompas. In case Oncokompas was not adopted, any subsequent requests of individual HCPs of these hospitals to implement Oncokompas individually were referred to the HCPs/oncological committee who decided not (yet) to adopt Oncokompas.
3	Instructional meeting: more in-depth information about Oncokompas as well as an explanation on how to offer Oncokompas to patients.
4	HCPs were provided with educational materials on Oncokompas: a script containing general information about Oncokompas, goals of the implementation, manuals on working with Oncokompas, screenshots of Oncokompas, and a frequently asked questions section (for patients and HCPs). A public website was also available with relevant information.
5	HCPs received promotional business cards of Oncokompas to hand out to patients.
6	Support and technical assistance for HCPs and patients were centralized and available through a helpdesk, operated by the Dutch Federation of Cancer Patient Societies.
7	Via the health insurance company, full reimbursement for all cancer survivors was assured. A prerequisite for reimbursement was that Oncokompas was to be offered by the HCP.
8	Progress in implementation was closely monitored and stimulated through an implementation advisory committee.

### Outcome measures

Adoption rate was calculated as the number of hospitals that adopted Oncokompas (agreed to start implementation) divided by the total number of eligible Dutch hospitals. Reasons for not adopting were explored based on minutes of meetings and e-mail conversations.

In the hospitals that adopted Oncokompas, all HCPs who obtained a HCP account for Oncokompas, automatically received an e-mail inviting them to complete an online questionnaire, 3 months after receiving the HCP account. This questionnaire consisted of study specific items and the MIDI questionnaire. The study specific items included work-related items (profession: medical specialist, nurse (specialist), (physician-)assistant, other) and the number of new patients they see each year. These were followed by the following items: (1) How many patients were offered Oncokompas?; (2) How many patients wanted to discuss the results of Oncokompas during a follow-up consult?; (3) How many patients brought a (digital) copy of their Oncokompas dossier to the consultation? (these questions had five answer categories: “1–5 patients,” “6–10 patients,” “11–50 patients,” “more than 50 patients,” “none”); (4) Did you offer Oncokompas to patients yourself? (yes, no); and (5) Why did you not offer Oncokompas? (multiple options allowed: “I don’t have the time to invite the patient to Oncokompas,” “Offering Oncokompas was done by somebody else,” “I forgot to register the patient for Oncokompas,” “I don’t endorse the use of Oncokompas,” “I don’t endorse the content of Oncokompas,” and a free text option). In case of non-response, personal reminders (at most three) followed by e-mail and telephone.

The MIDI questionnaire consists of 65 items addressing 29 determinants in four domains: the innovation itself (Oncokompas, eight items), the user (HCPs, 46 items), the organization (hospital, ten items), and the socio-political context (Dutch healthcare setting with accompanying laws and regulations, one item) [20]. These determinants may positively or negatively influence the implementation. For example, a low score on “professional obligation” in the context of this study means that an HCP perceives the goals that can be achieved with Oncokompas not as part of their job description, while a high score on, e.g., “correctness” means the HCP (strongly) perceives Oncokompas to be based on factual knowledge. For the current study, the MIDI was adapted to the context of Oncokompas, as is common practice when using the MIDI. Determinant 7, relevance for client, was for the purpose of this study divided into positive (“I think Oncokompas is suitable for my patients”) and negative relevance (“I think the use of Oncokompas is cumbersome for patients”). Determinant 10, professional obligation, consisted of a list of the core features of Oncokompas (which could be considered self-management behaviors an HCP could display), where HCPs are asked whether they think it is part of their job. See Appendix B for the complete questionnaire.

A higher mean score indicates that an HCP perceives this determinant less as a barrier to implement Oncokompas; higher scores are associated with higher expected levels of use [20].

### Statistical analysis

Adoption rate was calculated as the number of hospitals that adopted Oncokompas divided by the total number of eligible Dutch hospitals. Implementation rate was calculated as the number of HCPs in the adopting hospitals that reported that their patients were offered Oncokompas divided by the total number of HCPs in the adopting hospitals that responded to the online questionnaire.

Differences between implementers and non-implementers were assessed using Mann-Whitney *U* tests. Effect sizes were calculated via $r=z/\sqrt{n}$, where *z* is the standardized *U* statistic of the Mann-Whitney *U* test and *n* the total sample size. Because of the high number of determinants, significance was set at *p* ≤ 0.001 (two-tailed). Statistical analyses were performed using the Statistical Package for the Social Sciences (SPSS) version 24 (IBM Corp., Armonk, NY, USA).

## Results

### Adoption

**Fig. 2:**
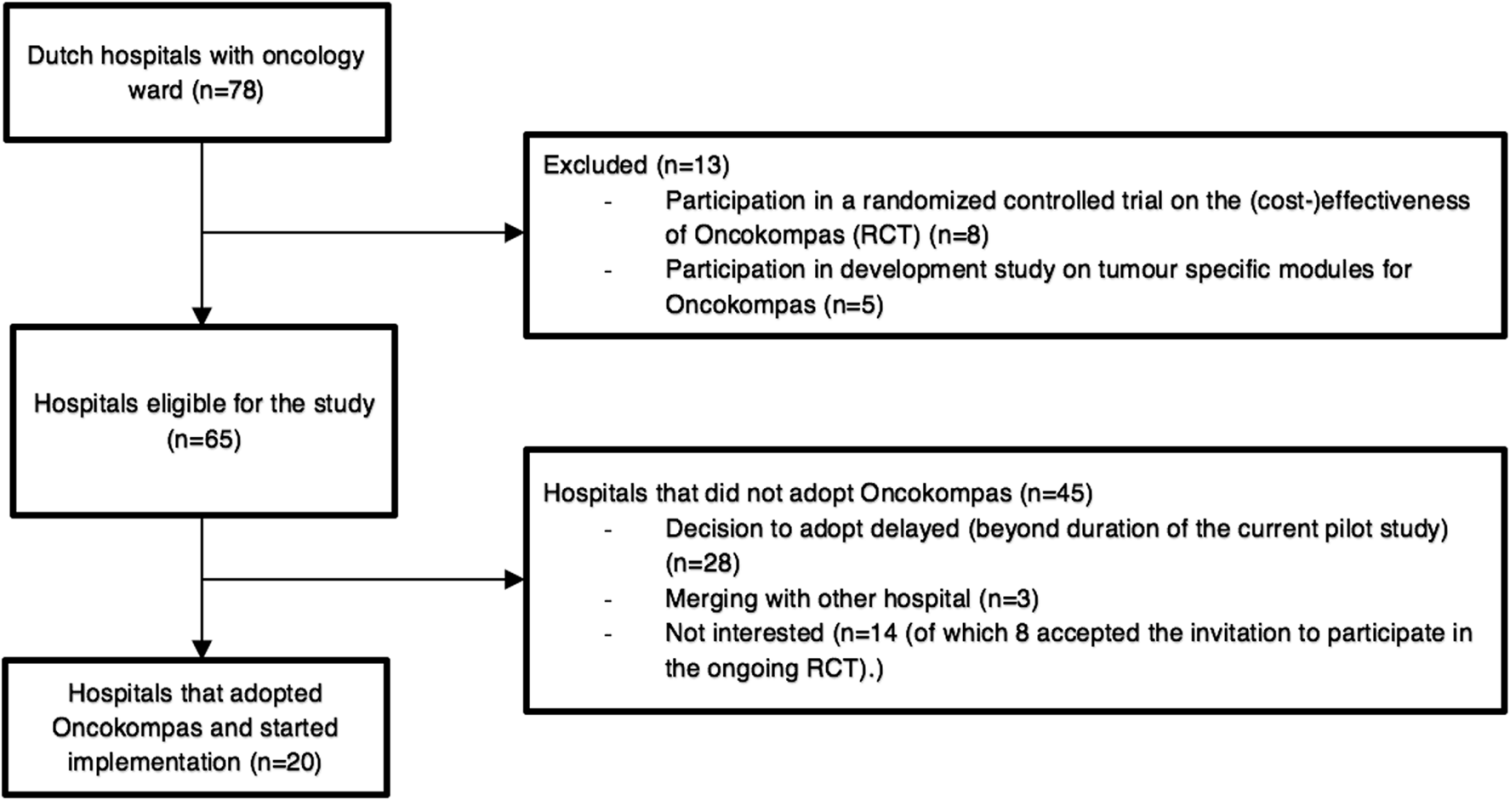
Flow chart of the adoption of Oncokompas

During this 1-year study, 20 out of 65 eligible hospitals adopted Oncokompas (adoption rate 31%). Various reasons for not (yet) adopting Oncokompas were mentioned. During the study, three hospitals were merging with another hospital and therefore postponed implementation of Oncokompas, 28 hospitals delayed the decision to implement Oncokompas beyond the duration of the study, and 14 hospitals decided not to adopt Oncokompas, of which eight hospitals accepted the invitation to participate in a randomized controlled trial on the (cost-)effectiveness of Oncokompas (see Fig. 2).

Some hospitals had implemented an alternative tool (e.g., the distress thermometer [24]), some wanted to wait for the results of the ongoing research on the (cost-)effectiveness of Oncokompas, and some perceived uncertainty about reimbursement of Oncokompas in the long run. Offering Oncokompas through an HCP instead of allowing survivors to register themselves was subject of critique for some HCPs, but not a reason for not adopting Oncokompas.

### Implementation

**Fig. 3:**
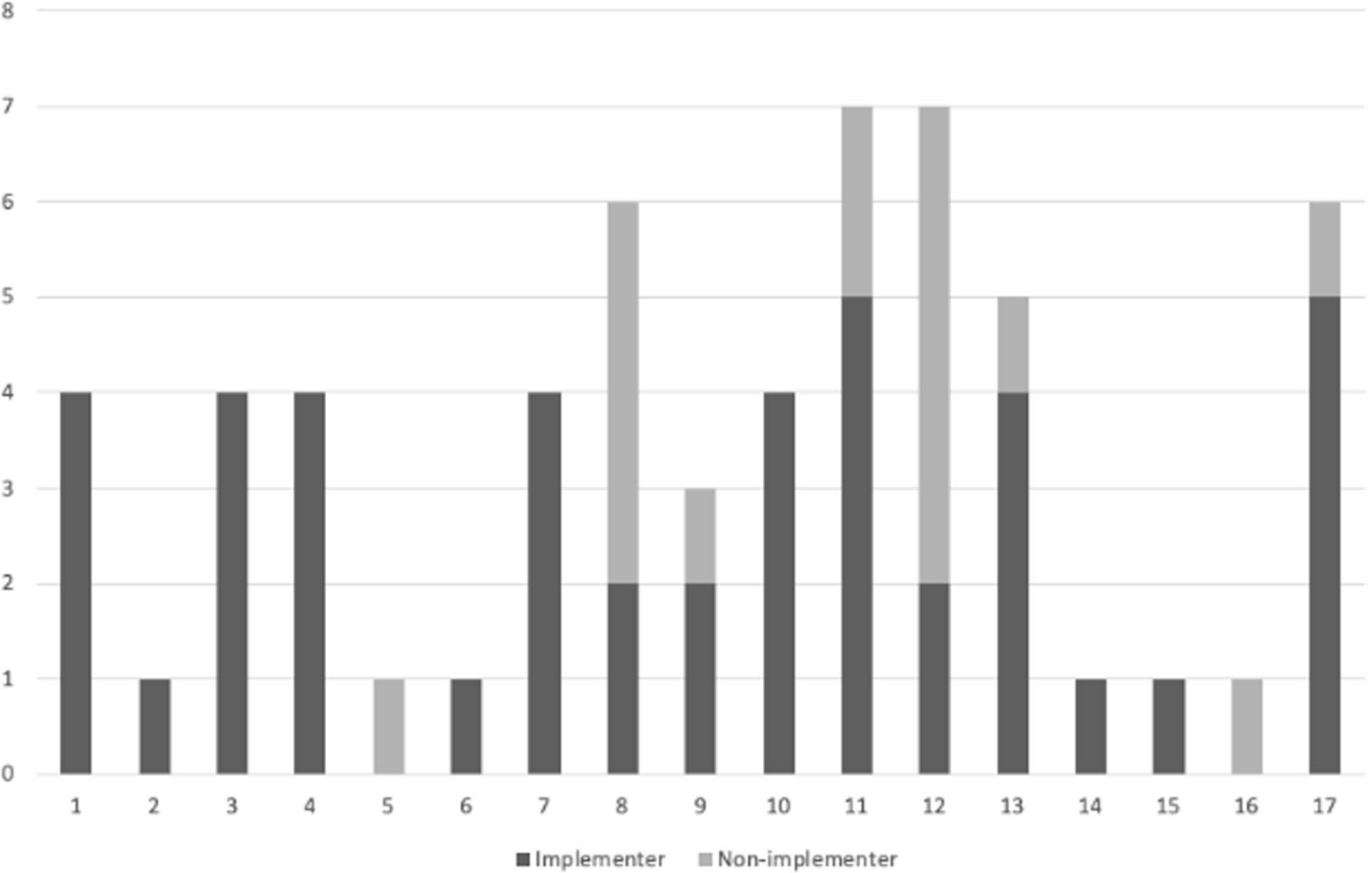
Number of implementers and non-implementers in each of the 17 adopting hospitals

Within the 20 hospitals that adopted Oncokompas, 205 HCPs were sent a questionnaire, of whom 72 responded (35%). HCPs were included who completed the work-related items and at least answered the items on innovation (Oncokompas) related MIDI determinants (*n* = 61) (Table 2). HCPs were mainly nurses (87%), and most provided cancer care to more than 50 new patients each year (82%). In total, 44 out of 61 HCPs (of 205 in total) responded that Oncokompas was offered to their patients: implementation rate 72%. There were no significant differences between implementers and non-implementers, with respect to profession or number of new patients per year. The 61 participants were spread over 17 hospitals, ranging from a total of 1 to 7 HCPs per hospital (Fig. 3).

**Table 2:** Results of implementation by healthcare professionals

	Total group (*N* = 61)	Implementers (*N* = 44)	Non-implementers (*N* = 17)
	*N* (%)	*N* (%)	*N* (%)
Profession^a^			
- Medical specialist	1 (1.6%)	1 (2.3%)	0
- Nurse	37 (60.7%)	27 (61.4%)	10 (58.8%)
- Nurse specialist	16 (26.2%)	13 (29.5%)	3 (17.6%)
- Support staff^b^	7 (11.5%)	3 (6.8%)	4 (23.5%)
Number of new patients each year^a^			
- 1–5	0	0	0
- 6–10	0	0	0
- 11–50	10 (16.4%)	6 (13.6%)	4 (23.5%)
- More than 50	50 (82.0%)	38 (86.4%)	12 (70.6%)
- None	1 (1.6%)	0	1 (5.9%)
Number of patients that Oncokompas was offered to.			
- 1–5	19	19 (43.2%)	NA
- 6–10	10	10 (22.7%)	NA
- 11–50	11	11 (25.0%)	NA
- More than 50	4	4 (9.1%)	NA
- None	17	0	NA
Main reasons for (occasionally) not offering Oncokompas (multiple answers allowed)			
- No time to offer Oncokompas	6 (9.8%)	2 (4.5%)	4 (23.5%)
- Was done by somebody else (e.g., nurse or MD)	9 (14.8%)	7 (15.9%)	2 (11.8%)
- Forgot to register the patient	24 (39.3%)	20 (45.5%)	4 (23.5%)
- I do not endorse the use of Oncokompas	1 (1.6%)	0	1 (5.9%)
- I do not endorse the content of Oncokompas	4 (6.6%)	3 (6.8%)	1 (5.9%)
Custom reasons for not offering Oncokompas			
- Mainly patients in palliative care	5 (8.2%)	1 (2.3%)	4 (23.6%)
- Patients do not have access to the internet	4 (6.6%)	4 (9.1%)	0
- Too difficult (for patient)	3 (4.9%)	2 (4.5%)	1 (5.9%)
- Unfamiliarity with Oncokompas	4 (6.6%)	2 (4.5%)	2 (11.8%)
Number of patients with whom Oncokompas was discussed during follow-up consult			
- 1–5	21	19 (43.2%)	NA
- 6–10	4	4 (9.1%)	NA
- 11–50	3	3 (6.8%)	NA
- None	33	18 (40.9%)	NA
Number of patients that brought a print or digital copy of their Oncokompas dossier			
- 1–5	3	3 (6.8%)	NA
- None	58	41 (93.2%)	NA

NA: not applicable

^a^No significant differences were found between groups

^b^Medical secretary, physician assistant

HCPs were asked for reasons for (occasionally) not offering Oncokompas. Not offering Oncokompas was mostly related to forgetting to register the patient in Oncokompas (39%) or because Oncokompas was offered by somebody else in their organization (15%). Some HCPs mentioned that Oncokompas is not yet suitable for their patient group, which consisted mainly of patients in palliative care (the version of Oncokompas available during the pilot implementation study was developed for cancer survivors). General perception about lack of digital skills among elderly patients (70+) was also mentioned as a reason not to offer Oncokompas.

Oncokompas was discussed in follow-up consults among 59% of the HCPs and 7% of the HCPs reported that 1–5 survivors brought along a (digital) copy of their Oncokompas dossier.

### Determinants of implementation

Implementers, in general, scored higher on the MIDI determinants than non-implementers (Table 3). Significant differences (*p* ≤ .001) between both groups were found in two determinants related to the innovation (Oncokompas), and four determinants related to the user (HCP) (Table 3). Compared to implementers, non-implementers scored significantly lower (perceived more barriers) on procedural clarity (*r* = 0.54, *p* < .001) and found offering Oncokompas more complex than implementers (complexity, *r* = 0.40, *p* = .002) (innovation related determinants). They scored lower (perceived more barriers) on importance of outcome expectations (*r* = 0.46, *p* < .001), professional obligation (*r* = 0.52, *p* < .001), social support (r = 0.46, *p* < .001), and self-efficacy (*r* = 0.44, *p* < .001) (user-related determinants).

**Table 3:** Perception of determinants of implementation by implementers and non-implementers

	Total mean (sd)	*N*	Implementers mean (sd)	Non-implementers mean (sd)	Mann-Whitney *U* statistic	*Z* score	Effect size *r*	*p*
Determinants associated with the innovation								
1. Procedural clarity	3.89 (1.10)	61	4.23 (.89)	3.00 (1.12)	129.5	4.228	0.54	< 0.001
2. Correctness	3.66 (.60)	61	3.77 (.61)	3.35 (.49)	239.0	2.448	0.31	0.014
3. Completeness	3.79 (.84)	61	3.93 (.76)	3.41 (.94)	256.5	2.081	0.26	0.037
4. Complexity^a^	3.48 (.92)	61	3.66 (.91)	3.00 (.79)	197.0	3.119	0.40	0.002
5. Compatibility	2.97 (.75)	61	3.05 (.78)	2.76 (.66)	294.0	1.456	0.18	0.146
6. Observability	2.34 (.83)	61	2.20 (.88)	2.71 (.59)	260.0	2.061	0.26	0.039
7a. Relevance for client	3.16 (.66)	61	3.25 (.69)	2.94 (.56)	271.5	1.881	0.24	0.060
7b. Negative relevance for client	2.72 (.61)	61	2.64 (.57)	2.94 (.66)	263.0	2.067	0.26	0.039
Determinants associated with the user (healthcare professional)								
8a. Personal benefits	2.64 (.66)	61	2.57 (.73)	2.82 (.46)	318.5	0.919	0.12	0.36
8b. Personal drawbacks	2.89 (.62)	61	2.88 (.67)	2.90 (.50)	345.5	0.496	0.06	0.62
9a. Outcome expectations (importance)	3.68 (.57)	60	3.85 (.53)	3.25 (.43)	158.0	3.576	0.46	< 0.001
9b. Outcome expectations (probability)	3.50 (.51)	60	3.60 (.50)	3.22 (.42)	209.0	2.684	0.35	0.007
10. Professional obligation	4.03 (.63)	58	4.22 (.55)	3.49 (.54)	117.0	3.985	0.52	< 0.001
11. Client/patient satisfaction	3.35 (.61)	57	3.44 (.67)	3.07 (.27)	201.5	2.148	0.28	0.032
12. Client/patient cooperation	3.32 (.78)	57	3.35 (.87)	3.21 (.43)	216.0	0.814	0.11	0.42
13. Social support	3.41 (.58)	57	3.55 (.58)	3.00 (.35)	117.0	3.482	0.46	< 0.001
14. Descriptive norm^b^	3.67 (1.7)	57	3.95 (1.72)	2.79 (1.37)	181.5	2.267	0.30	0.023
15a. Subjective norm (normative beliefs)	3.29 (.54)	56	3.39 (.56)	3.00 (.38)	142.5	2.910	0.39	0.004
15b. Subjective norm (motivation to comply)	3.50 (.49)	56	3.55 (.50)	3.34 (.40)	210.5	1.610	0.21	0.107
16. Self-efficacy	3.46 (.59)	54	3.59 (.50)	3.03 (.67)	109.0	3.231	0.44	0.001
17. Knowledge	3.28 (.90)	54	3.46 (.81)	2.69 (.95)	137.5	2.860	0.39	0.004
18. Awareness of content of innovation^c^	2.87 (.74)	61	3.00 (.72)	2.53 (.72)	242.0	2.286	0.29	0.022
19. Formal ratification by management^d^	17 (31.5%)	54	13 (31.7%)	4 (30.8%)	264.0	0.063	0.0§	0.95
20. Replacement when staff leave	2.80 (.87)	51	2.69 (.89)	3.17 (.72)	177.0	1.409	0.20	0.16
21. Staff capacity	3.08 (.87)	51	3.18 (.85)	2.75 (.87)	172.5	1.449	0.20	0.15
22. Financial resources	2.98 (.58)	51	3.05 (.61)	2.75 (.45)	175.5	1.559	0.22	0.12
23. Time available	3.02 (.79)	51	3.03 (.84)	3.00 (.60)	221.0	0.315	0.04	0.75
24. Material resources and facilities	3.37 (.72)	51	3.46 (.76)	3.08 (.52)	151.5	2.022	0.28	0.043
25. Coordinator^d^	35 (67.3%)	52	27 (67.5%)	8 (66.7%)	238.0	0.053	0.01	0.96
26. Unsettled organization^d^	32 (62.7%)	51	26 (66,7%)	6 (50.0%)	195.0	1.034	0.14	0.30
27. Information accessible about use of the innovation	3.53 (.86)	51	3.62 (.82)	3.25 (.97)	177.5	1.502	0.21	0.13
28. Performance feedback	2.67 (.89)	51	2.67 (.98)	2.67 (.49)	234.0	0.000	0.00	1.00
Determinants associated with the socio-political context								
29. Legislation and regulations	3.25 (.66)	51	3.28 (.72)	3.17 (.39)	197.0	0.935	0.13	0.35

A higher mean score indicates that a healthcare professional perceives this determinant less as a barrier to implement (ranging from 1 to 5)

^a^Determinant 4 is reversed for readability (low score indicates high complexity)

^b^Determinant 14 has 7 answer options: (1) not a single colleague, (2) almost no colleagues, (3) a minority, (4) half, (5) a majority, (6) almost all colleagues, (7) all colleagues) (ranging from 1 to 7)

^c^Determinant 18 has 4 answer categories: (1) I’m not familiar with the content of Oncokompas, (2) I’m familiar with Oncokompas, but I haven’t gone through it (yet), (3) I’m familiar with Oncokompas and I’ve looked at the clickable demo, (4) I’m familiar with the innovation and I have gone through it completely) (ranging from 1 to 4)

^d^Determinants 19, 25, and 26 are yes/no questions (ranging 1–2), *N* (%) that reported “yes” is displayed

## Discussion

This study describes the first steps of implementing a web-based self-management tool “Oncokompas” in Dutch oncology settings. Adoption rate by hospitals was at 31%, and implementation rate in the adopting hospitals by HCPs was at 72%. However, when viewed in the context of the adoption rate, one could also argue that overall implementation rate was low. For instance, taking the total number of HCPs in the adopting hospitals (*n* = 205) as denominator, the implementation rate would be 21%. In any case, the results are in line with the finding that many new interventions fail to be widely adopted [25]. The results can also be viewed in light of Roger’s diffusion of innovation theory where the adoption rate follows an S-curve plotted over time (when looking at the complete lifecycle of an innovation) [26]. When viewing the adoption of Oncokompas in this light, one could say the innovators and early adopters (31%) are using Oncokompas at present, while the others (early and late majority and laggards) are still contemplating or waiting.

To be able to reach cancer patients, the multifaceted implementation strategy focused on oncology settings in hospitals, because that is the place where every new patient can potentially be reached. For that to happen, first, the hospitals had to adopt Oncokompas, which is a challenge, because the organization of oncological care in hospitals is multidisciplinary and complex. Even though all stakeholders, including HCPs, were involved in every step of the development of Oncokompas [6, 11, 13], this does not guarantee immediate adoption and implementation. Several reasons were mentioned by hospitals for not adopting Oncokompas, such as a lack of evidence regarding (cost-)effectiveness and uncertainty about reimbursement by the health insurance company in the long-term.

Another barrier to adopt Oncokompas relates to the concept of self-management: some hospitals questioned whether access to an online self-management tool should be provided through a hospital or directly to patients themselves. More research is needed on the organization of self-management (support) in cancer care [27], but a key aspect in providing this support is cooperation between health services [28]. Also, in a previous qualitative study exploring HCP’s perspective regarding Oncokompas implementation, we found that all participants indicated that when Oncokompas was to be implemented in daily clinical practice, it should be offered to survivors through a clinical procedure in a care pathway [11].

Implementation by HCPs in the hospitals that adopted Oncokompas was at 72% and related primarily to innovation-related and user-related determinants. Procedural clarity was lower for those HCPs who did not implement Oncokompas, indicating more training or supportive material is needed. This finding could be an indication of a bad fit with procedures or organization of cancer care pathways, which could lead to delays in implementation [29], although the MIDI determinant “compatibility” in the current study was not significantly different between the two groups. This is important, because if (part of) the intervention is perceived as easy to implement, this will result in higher levels of implementation [30].

HCPs who did not offer Oncokompas also experienced less social support from management, colleagues, the helpdesk, and the Oncokompas team. Studies have shown that support from management is an important facilitator [31, 32]. In the present study, we generally tried to apply a top-down approach through the oncological committees. This often resulted in the committee deciding to first start in a single department or a single HCP implementing Oncokompas, which perhaps contributed towards a feeling of isolation for these HCPs and a perception of less social support.

Self-reported self-efficacy was significantly lower among those who did not offer Oncokompas, which is in line with findings that a higher self-efficacy is associated with higher levels of self-management support [33]. Low confidence is also associated with greater hesitance towards implementing innovations, which in turn could lead to lower implementation levels [34]. The difference in self-efficacy might also be related to social support. If a group as a whole is working towards implementation, perceived collective efficacy might positively interact with individual self-efficacy [35] (MIDI determinant descriptive norm). This in turn calls for central coordination and formal ratification by management (both MIDI determinants) when implementing an innovation. Additionally, taking a top-down approach and implementing an innovation hospital wide, involving all relevant staff, is considered to be crucial for successful implementation and integration.

Interestingly though, the two groups did not differ significantly on organizational related determinants of the MIDI, but one third reported there was no formal ratification by management, while previous studies have shown this to be an important factor [36, 37].

Quantitative insights into implementation of innovation in supportive cancer care are still relatively scarce, and the current study adds to this new body of research. There was however no qualitative follow-up (e.g., interviews), which is common in implementation process studies. Although we believe we captured many essential elements, this could have provided additional insights, such as a deeper understanding of why implementers generally score MIDI determinants higher than non-implementers. The cross-sectional design of the current study also limits the amount of relevant information that can be captured, as implementation is a process that develops over time, strongly influenced by current circumstances. Furthermore, the instructional meeting that was organized in the adopting hospitals was not attended by all HCPs involved, which could have had an effect on the implementation rate. Effects of training were not evaluated in the current study, but this exposure to education on working with the intervention should be captured in future studies. We defined implementers as those HCPs that followed the three steps involved in implementing Oncokompas, but this was based on self-reported answers by HCPs. Future studies should more thoroughly assess the level of implementation of the different components of interventions (and steps involved for the HCP) and measure if these are delivered as intended.

The response rate was low, which we tried to address beforehand by making the questionnaire as short as possible, leaving out demographic variables, such as sex and age. The questionnaire was available online, which might increase response [38]. There could also be a bias in the group of participants, as HCPs that did not implement Oncokompas as intended, were probably less likely to participate in this study.

Future research should aim to capture as many processes and perceptions as possible to be able to assess as much (underling) processes as possible.

The current dissemination strategy of offering Oncokompas through an HCP could be transformed in such a way that allows people to independently register for Oncokompas, but that would also require a shift in the way of thinking among healthcare insurance companies in order to secure reimbursement in order to sustain Oncokompas.

## Conclusion

During this 1-year study, nationwide adoption rate of Oncokompas was at 31% at the end of this study and subsequent implementation rate within this study was at 73%. Comparing those HCPs who did and did not implement Oncokompas, the groups differed significantly on innovation-related (procedural clarity, complexity) and user-related determinants (importance of outcome expectations, professional obligation, social support, and self-efficacy). Both groups encountered barriers concerning organization-related determinants. The results of this study contribute to further optimize interventions and strategies to adopt and implement (online) self-management applications in cancer care.

## Electronic supplementary material

ESM 1 (DOCX 10191 kb)

ESM 2 (DOCX 31 kb)

## References

[1] Lorig KR, Sobel DS, Ritter PL (2001). Effect of a self-management program on patients with
chronic disease. Eff Clin Pract.

[2] Boogaard L, Gater L, Mori M, Trincao A, Smith-Turchyn J (2016). Efficacy of self-management programs in managing side
effects of breast cancer: a systematic review and meta-analysis of randomized
control trials. Rehabil Oncol.

[3] Slev VN, Mistiaen P, Pasman HRW, Leeuw IMVD, Uden-Kraan CF, Francke AL (2016). Effects of eHealth for patients and informal
caregivers confronted with cancer: a meta-review. Int J Med Inform.

[4] van de Poll-Franse LV, van Eenbergen MCHJ (2008). Internet use by cancer survivors: current use and
future wishes. Support Care Cancer.

[5] Institute of Medicine (U.S.) (2003) Committee on identifying priority areas for quality improvement. In: Adams K, Corrigan J (eds) Priority areas for national action: transforming health care quality. Washington (DC): National Academies Press 25057643

[6] Duman-Lubberding S, van Uden-Kraan CF, Jansen F (2016). Feasibility of an eHealth application “OncoKompas” to
improve personalized survivorship cancer care. Support Care Cancer.

[7] Kim AR, Park H-A (2015). Web-based self-management support interventions for
cancer survivors: a systematic review and meta-analyses. Stud Health Technol Inform.

[8] Kim SH, Kim K, Mayer DK (2017). Self-management intervention for adult Cancer
survivors after treatment: a systematic review and meta-analysis. Oncol Nurs Forum.

[9] Verwijsgids Kanker [Internet]. [cited 2018 Jul 3]. Available from: https://www.verwijsgidskanker.nl/

[10] Lubberding S, van Uden-Kraan CF, Te Velde EA (2015). Improving access to supportive cancer care through an
eHealth application: a qualitative needs assessment among cancer
survivors. J Clin Nurs.

[11] Duman-Lubberding S, van Uden-Kraan CF, Peek N (2015). An eHealth application in head and neck cancer
survivorship care: health care professionals’ perspectives. J Med Internet Res.

[12] van Gemert-Pijnen JEWC, Nijland N, van Limburg M (2011). A holistic framework to improve the uptake and impact
of eHealth technologies. J Med Internet Res.

[13] Melissant HC, Verdonck-de Leeuw IM, Lissenberg-Witte BI, Konings IR, Cuijpers P, Van Uden-Kraan CF (2018) ‘Oncokompas’, a web-based self-management application to support patient activation and optimal supportive care: a feasibility study among breast cancer survivors. Acta Oncol (Madr) 57(7):924–34 10.1080/0284186X.2018.1438654 29451059

[14] Wiggers A-M, Vosbergen S, Kraaijenhagen R (2013). Changes in the cardiac rehabilitation workflow process
needed for the implementation of a self-management system. Stud Health Technol Inform.

[15] May C, Finch T (2009). Implementing, Embedding, and integrating practices: an
outline of normalization process theory. Sociology.

[16] Damschroder LJ, Aron DC, Keith RE, Kirsh SR, Alexander JA, Lowery JC (2009). Fostering implementation of health services research
findings into practice: a consolidated framework for advancing implementation
science. Implement Sci.

[17] Harvey J, Dopson S, McManus RJ (2015). Factors influencing the adoption of self-management
solutions: an interpretive synthesis of the literature on stakeholder
experiences. Implement Sci.

[18] Geerligs L, Rankin NM, Shepherd HL, Butow P (2018). Hospital-based interventions: a systematic review of
staff-reported barriers and facilitators to implementation
processes. Implement Sci.

[19] Grol R, Wensing M (2004). What drives change? Barriers to and incentives for
achieving evidence-based practice. Med J Aust.

[20] Fleuren MAH, Paulussen TGWM, Van Dommelen P (2014). Towards a Measurement Instrument for Determinants of
Innovations. Int J Qual Health Care.

[21] van der Hout A, van Uden-Kraan CF, Witte BI (2017). Efficacy, cost-utility and reach of an eHealth
self-management application “Oncokompas” that helps cancer survivors to obtain
optimal supportive care: study protocol for a randomised controlled
trial. Trials.

[22] Medical devices—European Commission [Internet]. [cited 2018 Apr 17]. Available from: https://ec.europa.eu/growth/sectors/medical-devices

[23] Waltz TJ, Powell BJ, Matthieu MM, Damschroder LJ, Chinman MJ, Smith JL, Proctor EK, Kirchner JAE (2015). Use of concept mapping to characterize relationships
among implementation strategies and assess their feasibility and importance:
results from the Expert Recommendations for Implementing Change (ERIC)
study. Implement Sci.

[24] Tuinman MA, Gazendam-Donofrio SM, Hoekstra-Weebers JE (2008). Screening and referral for psychosocial distress in
oncologic practice. Cancer.

[25] Gaglio B, Shoup JA, Glasgow RE (2013). The RE-AIM framework: a systematic review of use over
time. Am J Public Health.

[26] Rogers EM (2003). Diffusion of innovations.

[27] Slev VN, Pasman HRW, Eeltink CM (2017). Self-management support and eHealth for patients and
informal caregivers confronted with advanced cancer: an online focus group study
among nurses. BMC Palliat Care.

[28] Lawn S, Schoo A (2010). Supporting self-management of chronic health
conditions: common approaches. Patient Educ Couns.

[29] Belkora JK, Loth MK, Chen DF, Chen JY, Volz S, Esserman LJ (2008). Monitoring the implementation of consultation
planning, recording, and summarizing in a breast care center. Patient Educ Couns.

[30] Schmied V, Gribble K, Sheehan A, Taylor C, Dykes FC (2011). Ten steps or climbing a mountain: a study of
Australian health professionals’ perceptions of implementing the baby friendly
health initiative to protect, promote and support breastfeeding. BMC Health Serv Res.

[31] Greenhalgh T, Robert G, Macfarlane F (2004). Diffusion of innovations in service organizations:
systematic review and recommendations. Milbank Q.

[32] Liisa AA, Marja-Terttu T, Päivi Å-K, Marja K (2011). Health care personnel’s experiences of a bereavement
follow-up intervention for grieving parents. Scand J Caring Sci.

[33] van Hooft SM, Dwarswaard J, Bal R, Strating MM, van Staa AL (2016). What factors influence nurses’ behavior in supporting
patient self-management? An explorative questionnaire study. Int J Nurs Stud.

[34] Hughes R, Aspinal F, Addington-Hall JM, Dunckley M, Faull C, Higginson I (2004). It just didn’t work: the realities of quality
assessment in the English health care context. Int J Nurs Stud.

[35] Bandura A (2000). Exercise of human agency through collective
efficacy. Curr Dir Psychol Sci.

[36] Cummings GG, Estabrooks CA, Midodzi WK (2007). Influence of organizational characteristics and
context on research utilization. Nurs Res.

[37] Dannapfel P, Peolsson A, Nilsen P (2013). What supports physiotherapists’ use of research in
clinical practice? A qualitative study in Sweden. Implement Sci.

[38] Sax LJ, Gilmartin SK, Bryant AN (2003). Assessing response rates and nonresponse bias in web
and paper surveys. Res High Educ.

