# Supplementary material for: Adoption and implementation of a web-based self-management application “Oncokompas” in routine cancer care: a national pilot study
Source: Support Care Cancer. 2018 Dec 18;27(8):2911–20. doi: 10.1007/s00520-018-4591-5 (PMC6598735; doi:10.1007/s00520-018-4591-5)
Supplement: Supplementary file 1 — (DOCX 10191 kb) [file 520_2018_4591_MOESM1_ESM.docx]

**Multimedia Appendix 1. Screenshots of the Oncokompas flow**

The screenshots of the Oncokompas application in this document have been translated from Dutch to English. Original Dutch screenshots can be found below the English screenshots.


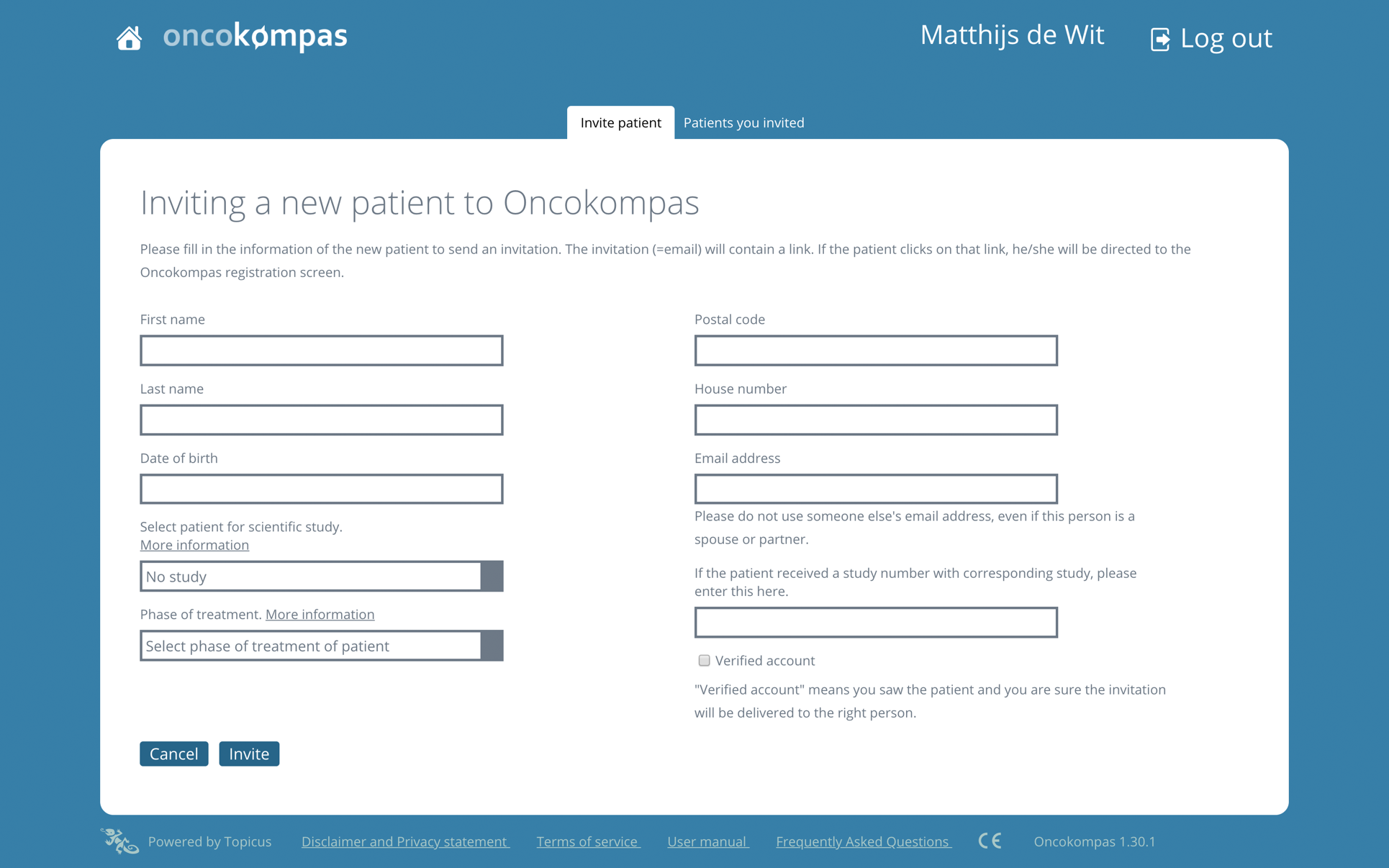


Screenshot 1. Health care provider portal. The patient receives an e-mail with a unique link to Oncokompas in order to create an account.


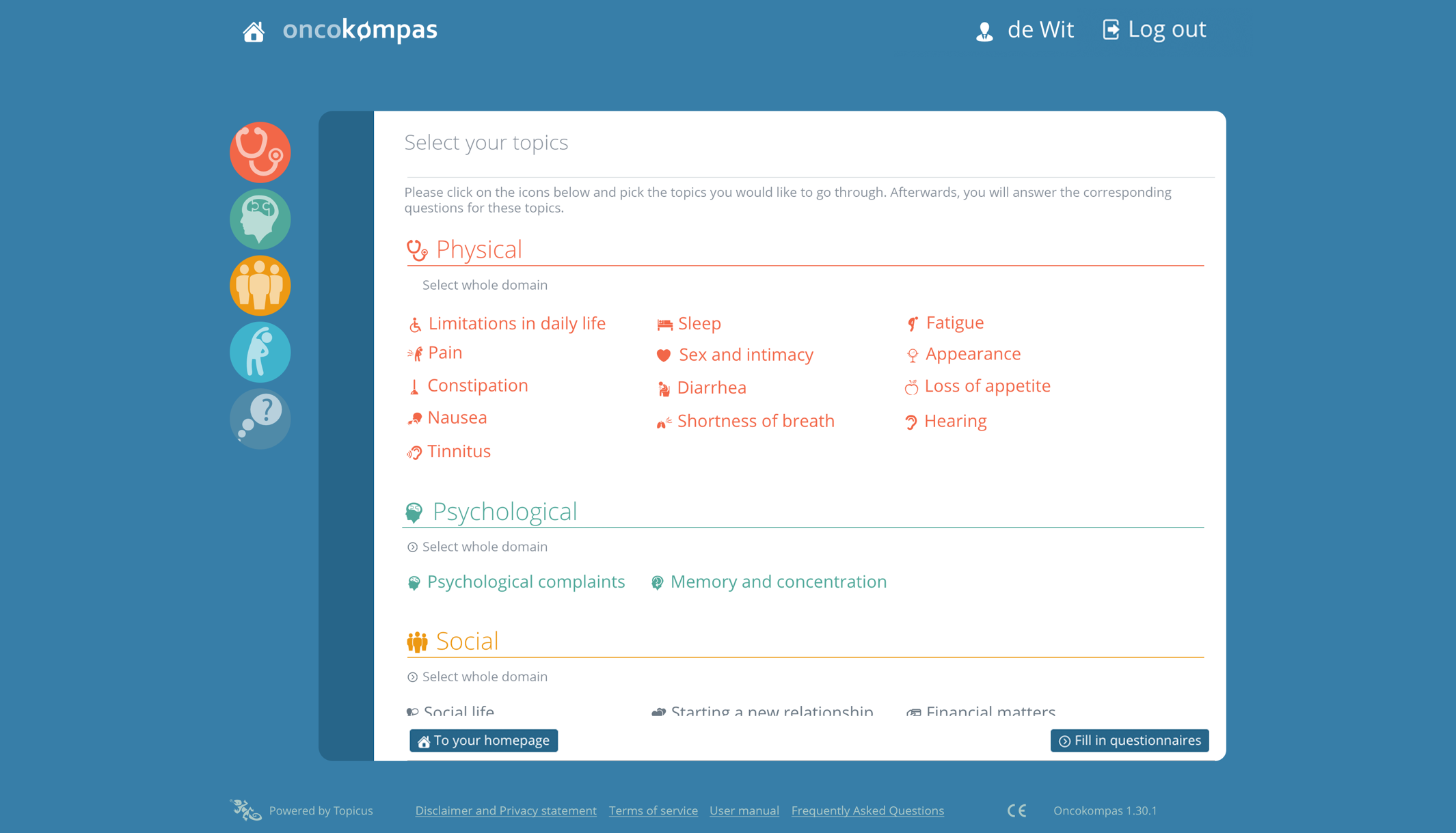


Screenshot 2. Patient portal. Selection of quality of life topics that are of interest to the patient.


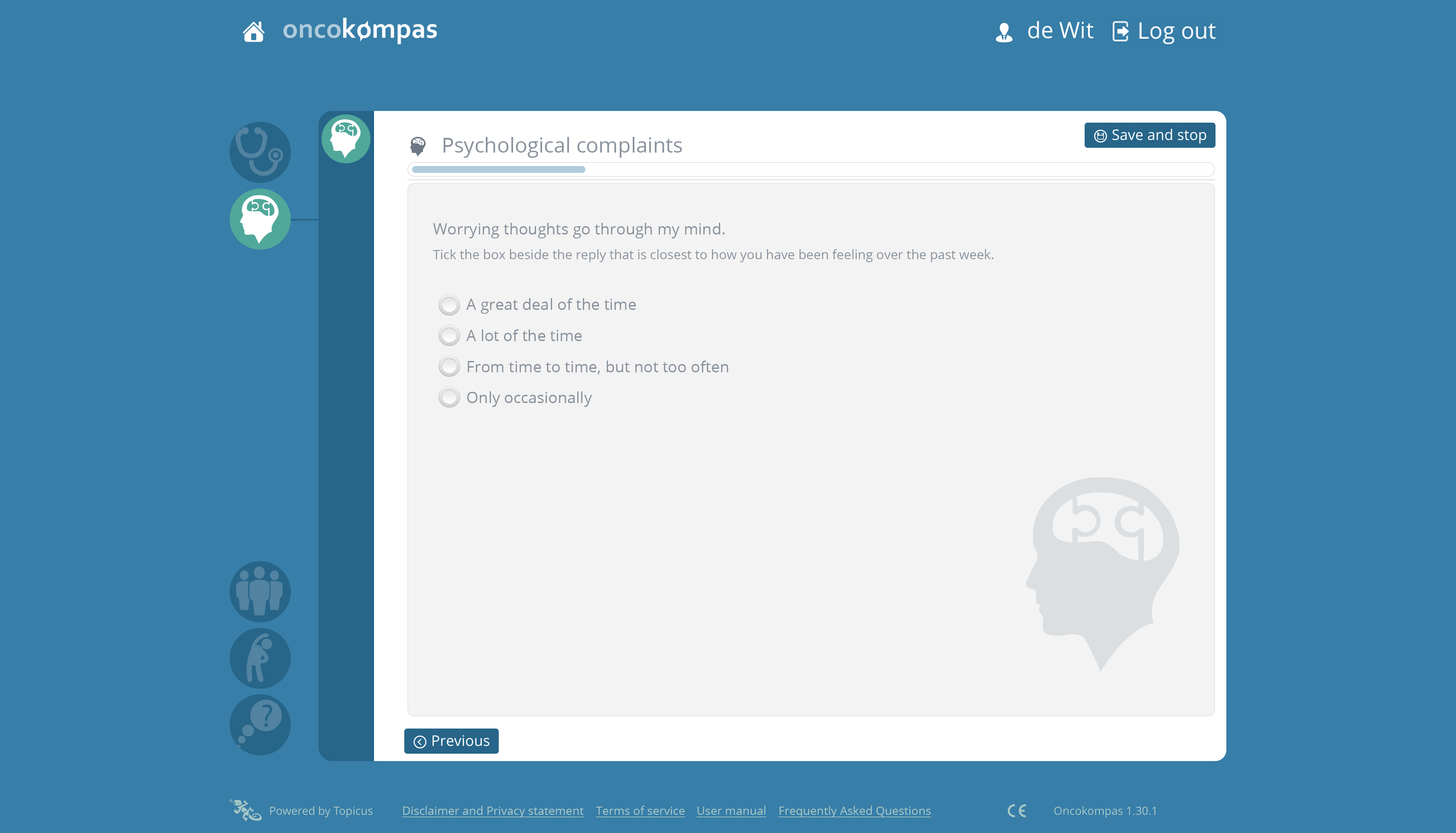


Screenshot 3. A question in the component ‘Measure’.


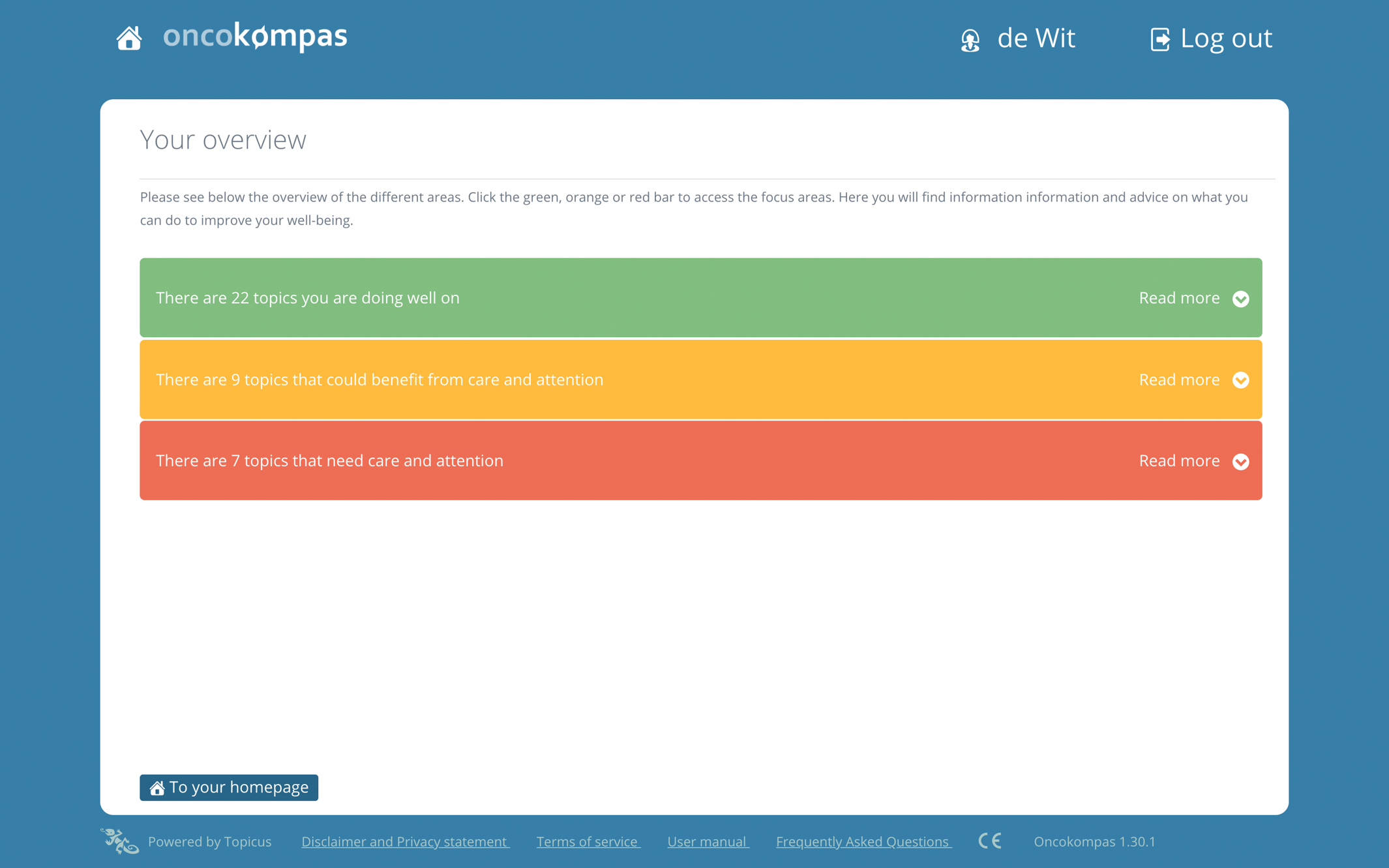


Screenshot 4. Feedback on the topics by means of a three-color system in the component ‘Learn’.


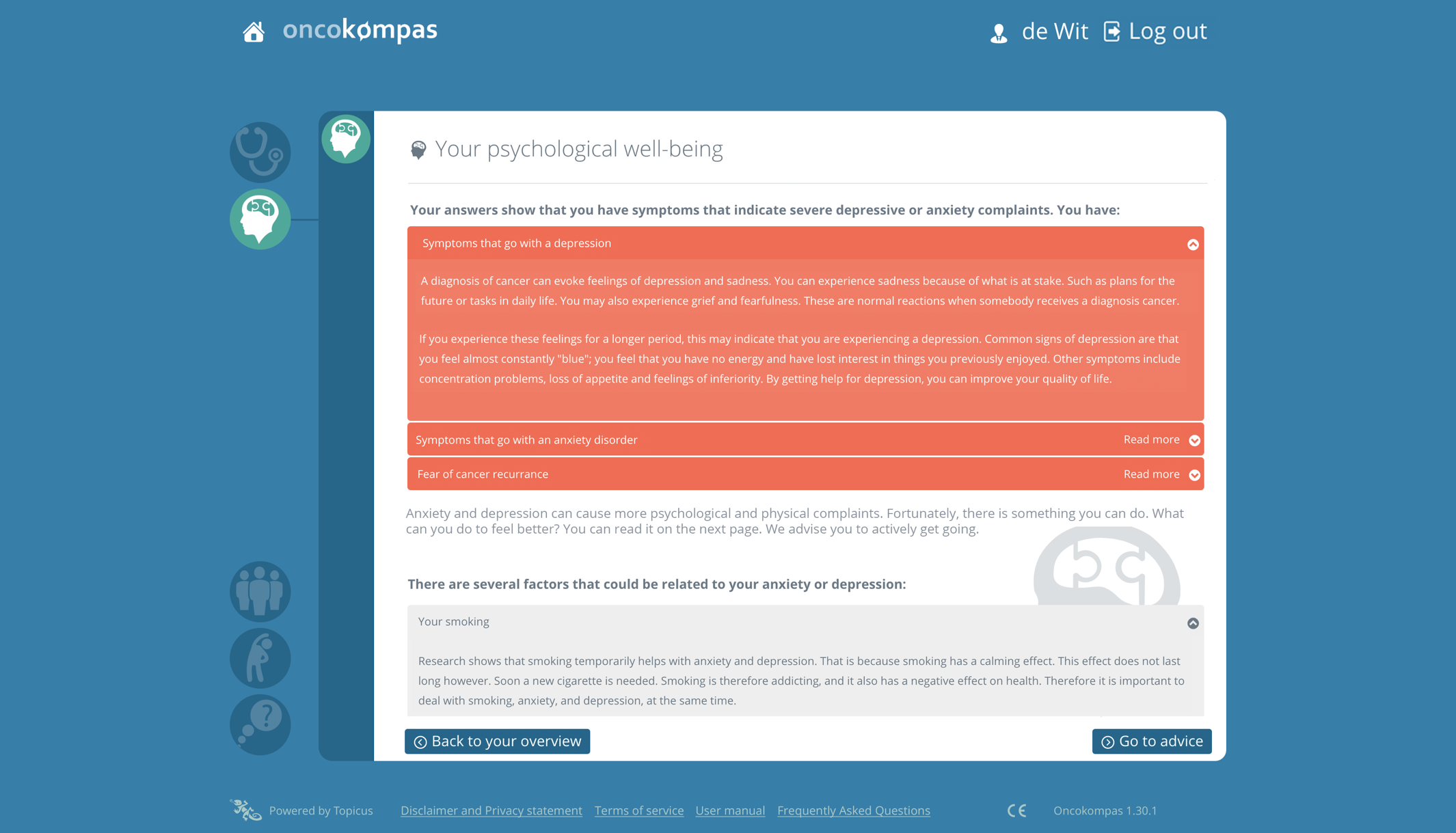


Screenshot 5. Personalized information on a topic in the component ‘Learn’.


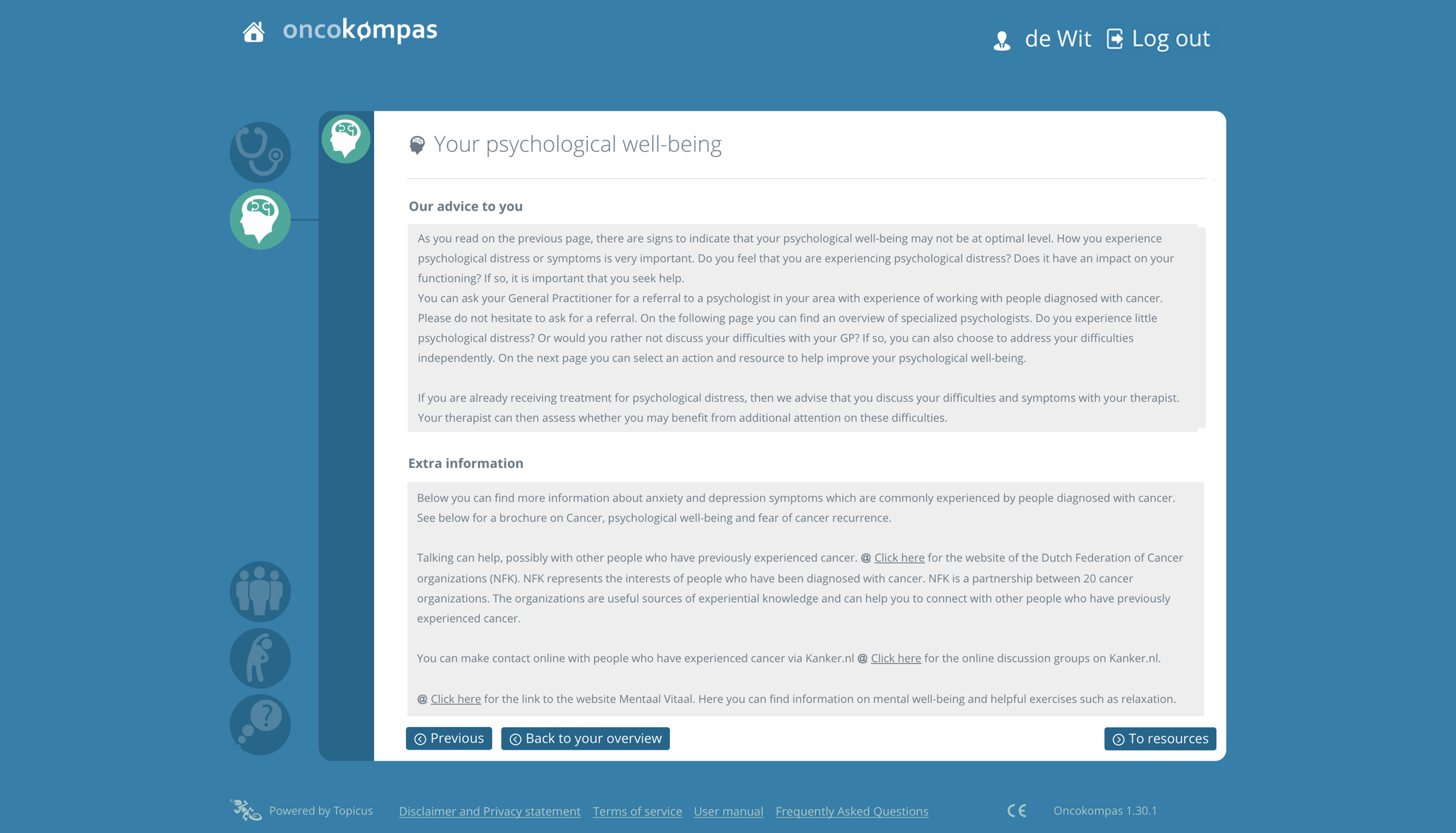


Screenshot 6. Tailored self-care advices in the component ‘Learn’.


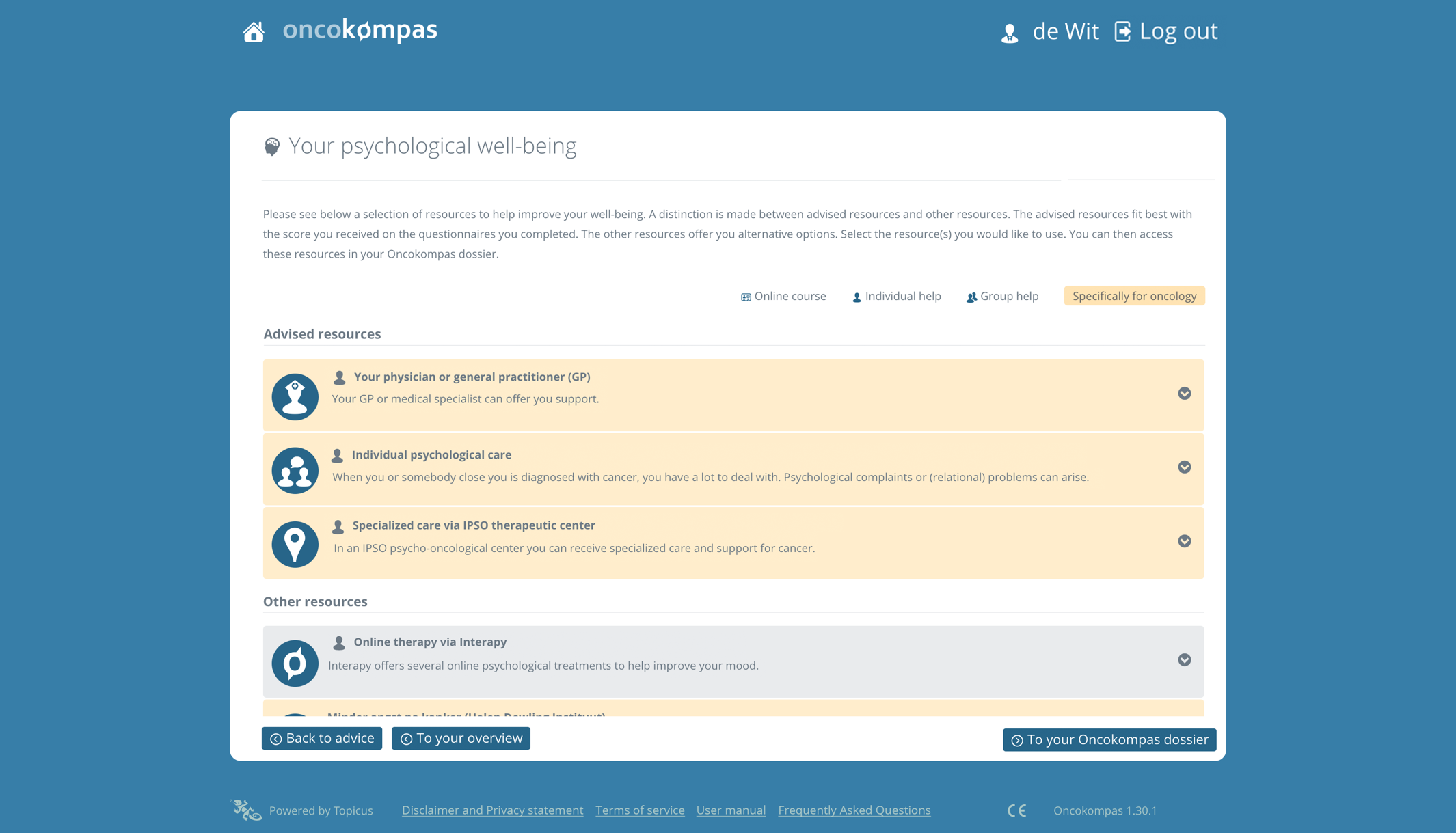


Screenshot 7. Overview of personalized supportive care options in the component ‘Act’.


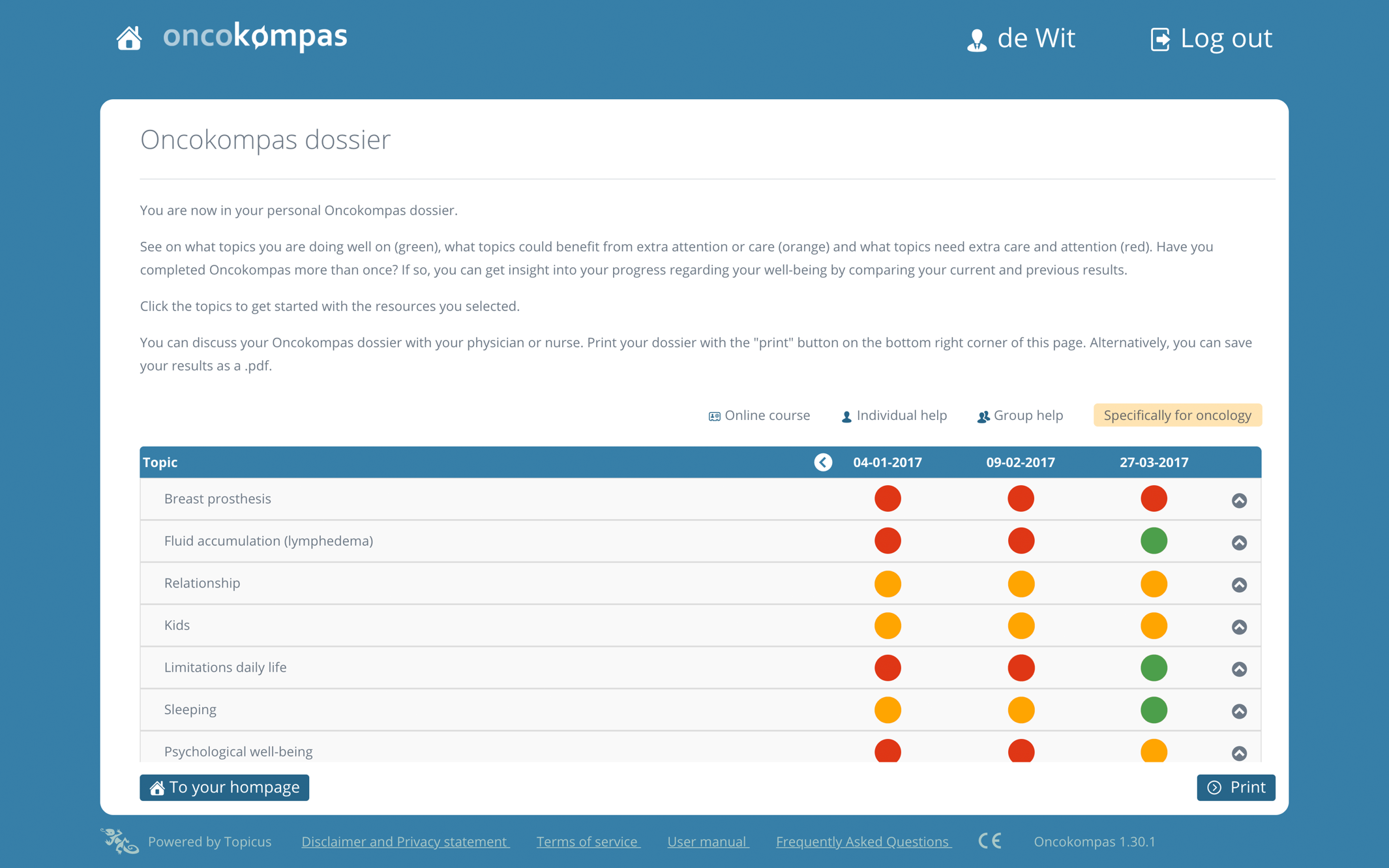


Screenshot 8. Oncokompas dossier function.

**Original Dutch screenshots**


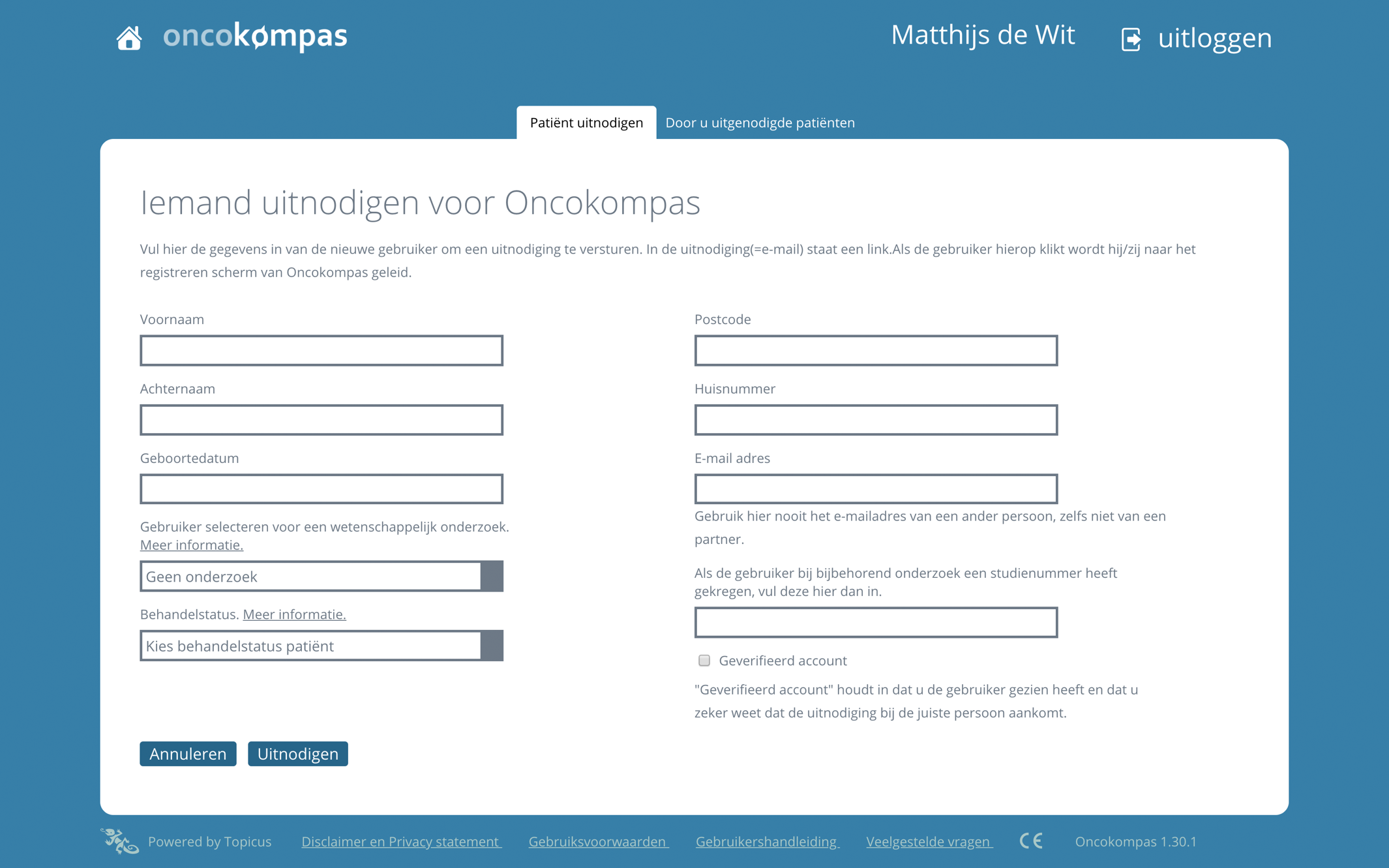


Screenshot 1. Health care provider portal. The patient receives an e-mail with a unique link to Oncokompas in order to create an account.


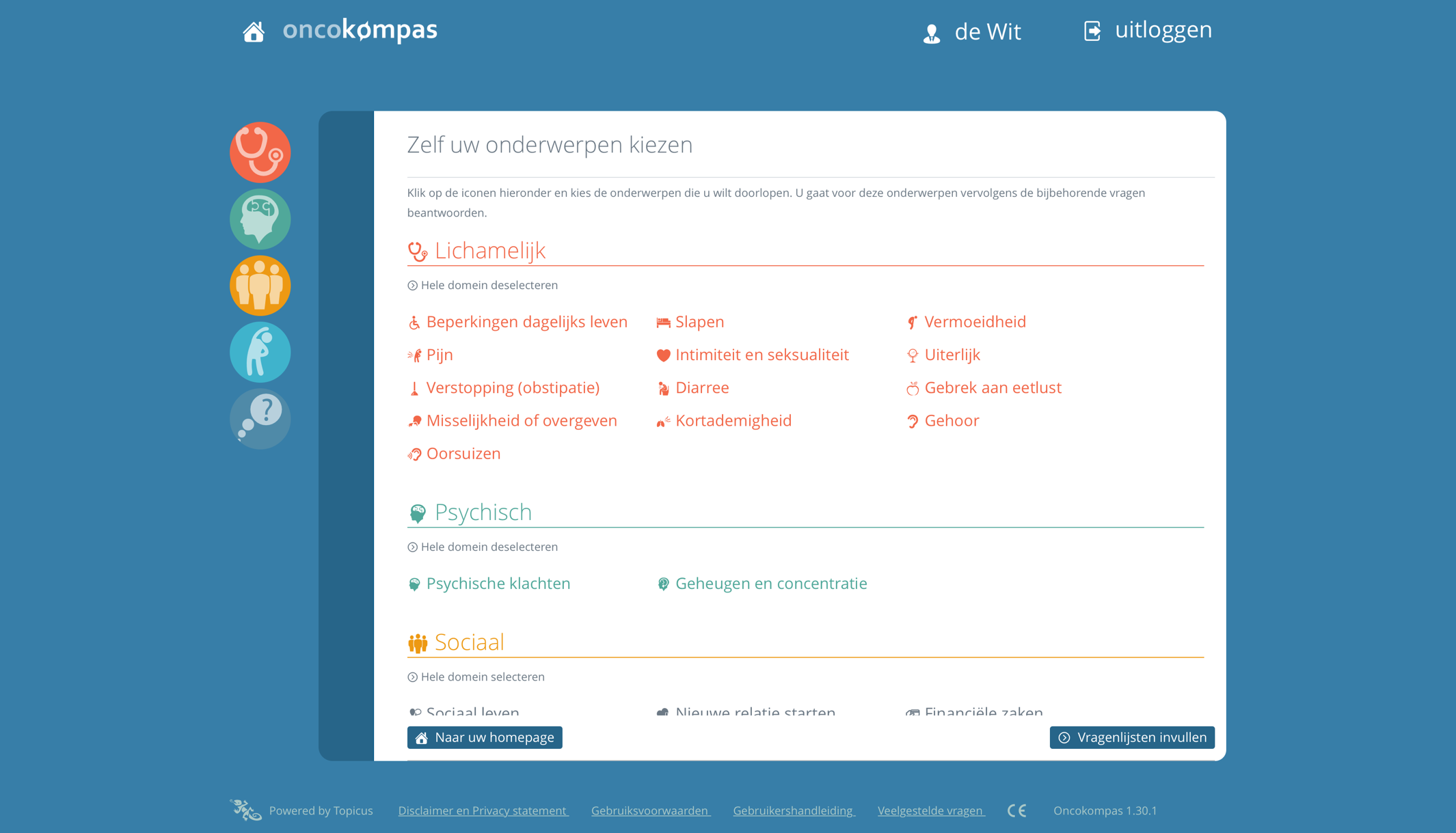


Screenshot 2. Patient portal. Selection of quality of life topics that are of interest to the patient.


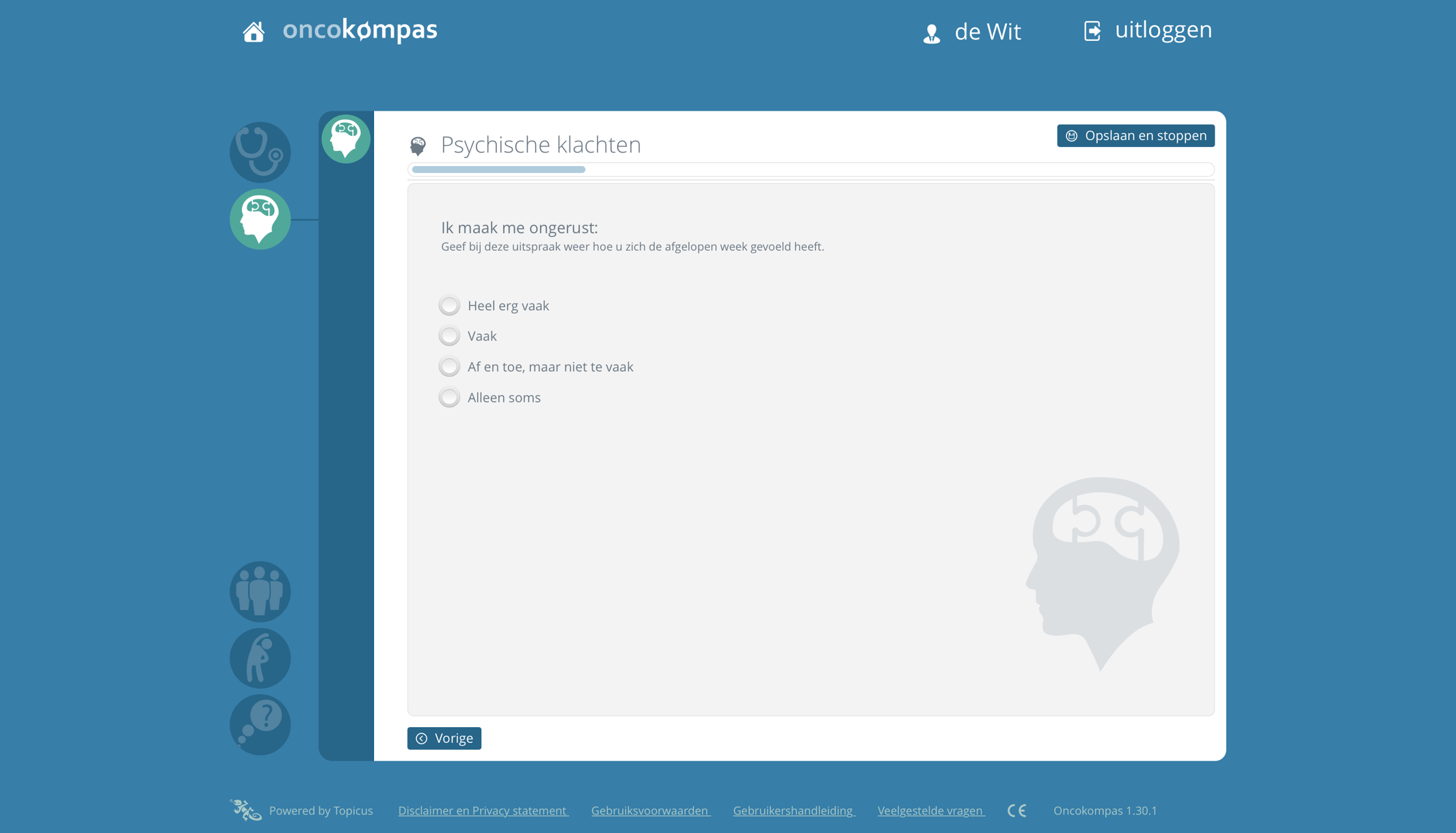


Screenshot 3. A question in the component ‘Measure’.


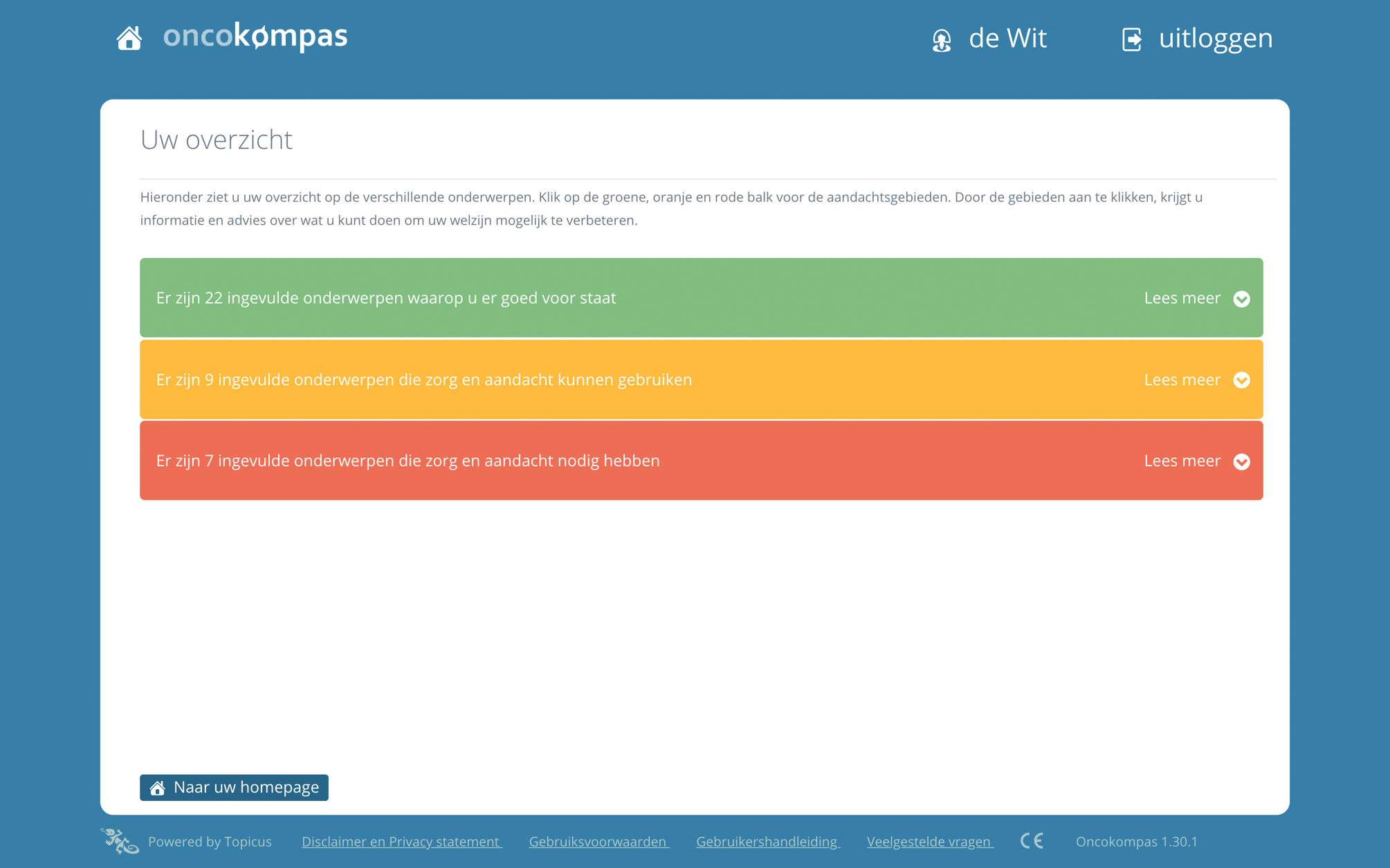


Screenshot 4. Feedback on the topics by means of a three-color system in the component ‘Learn’.


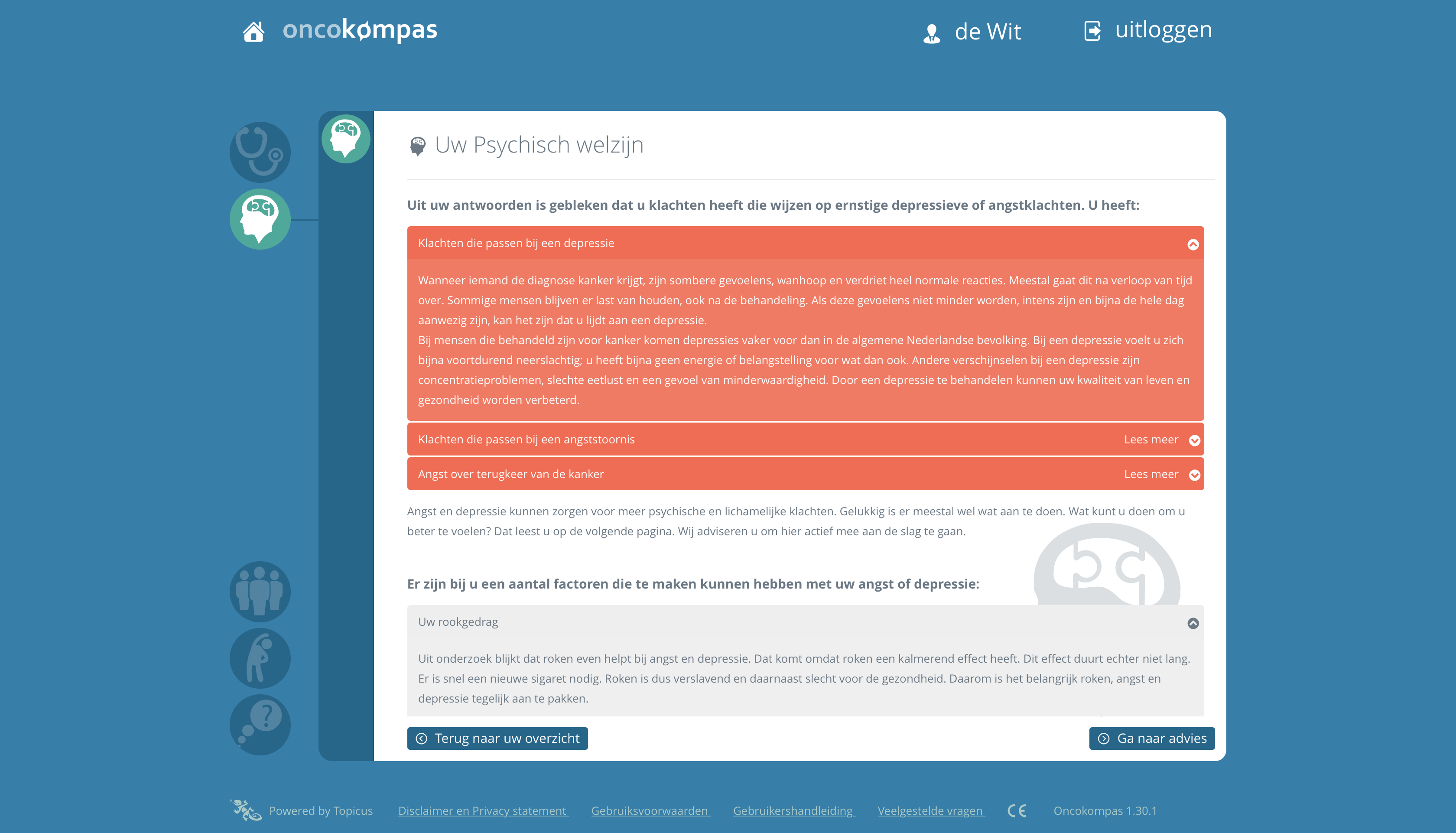


Screenshot 5. Personalized information on a topic in the component ‘Learn’.


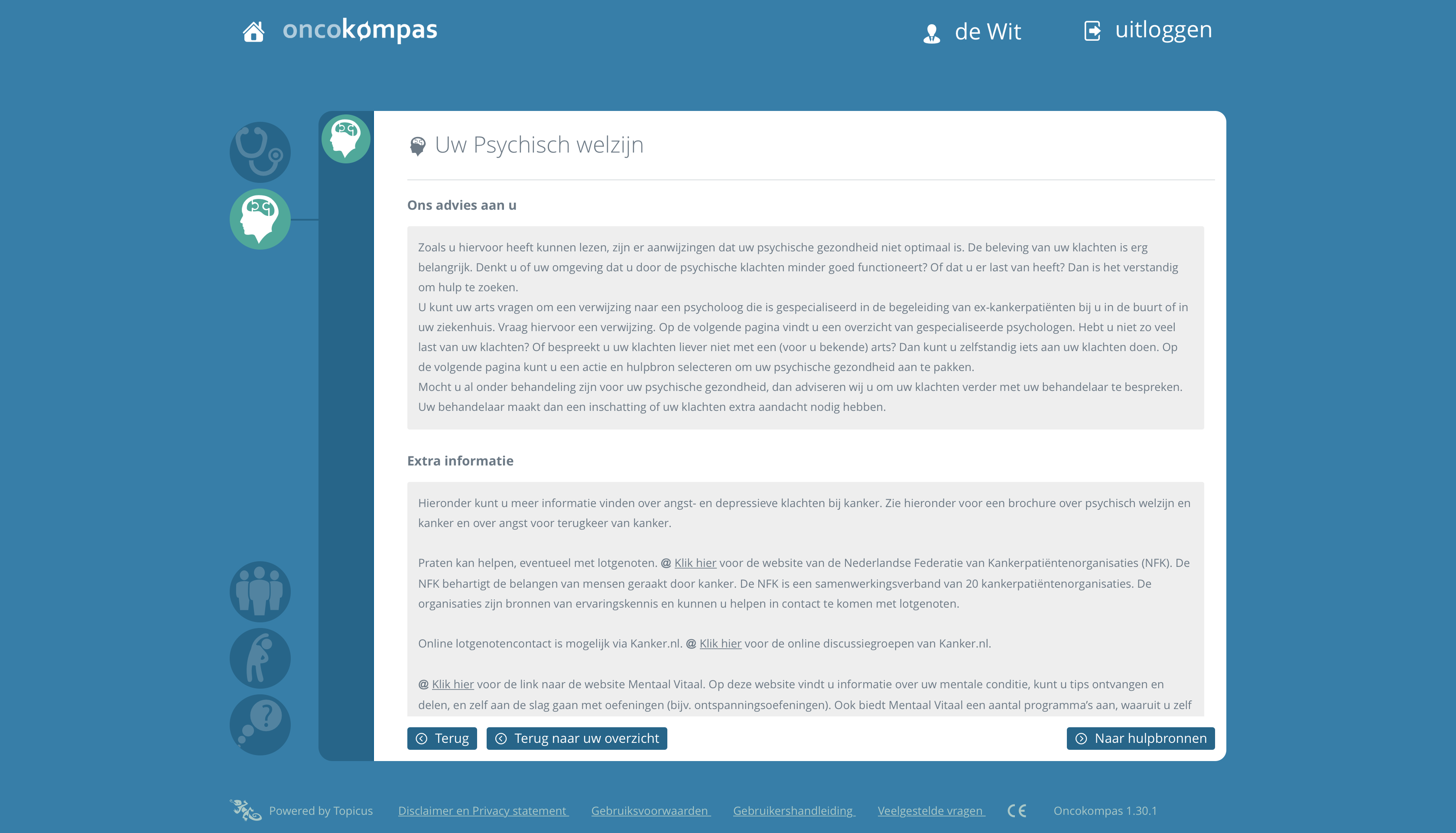


Screenshot 6. Tailored self-care advices in the component ‘Learn’.


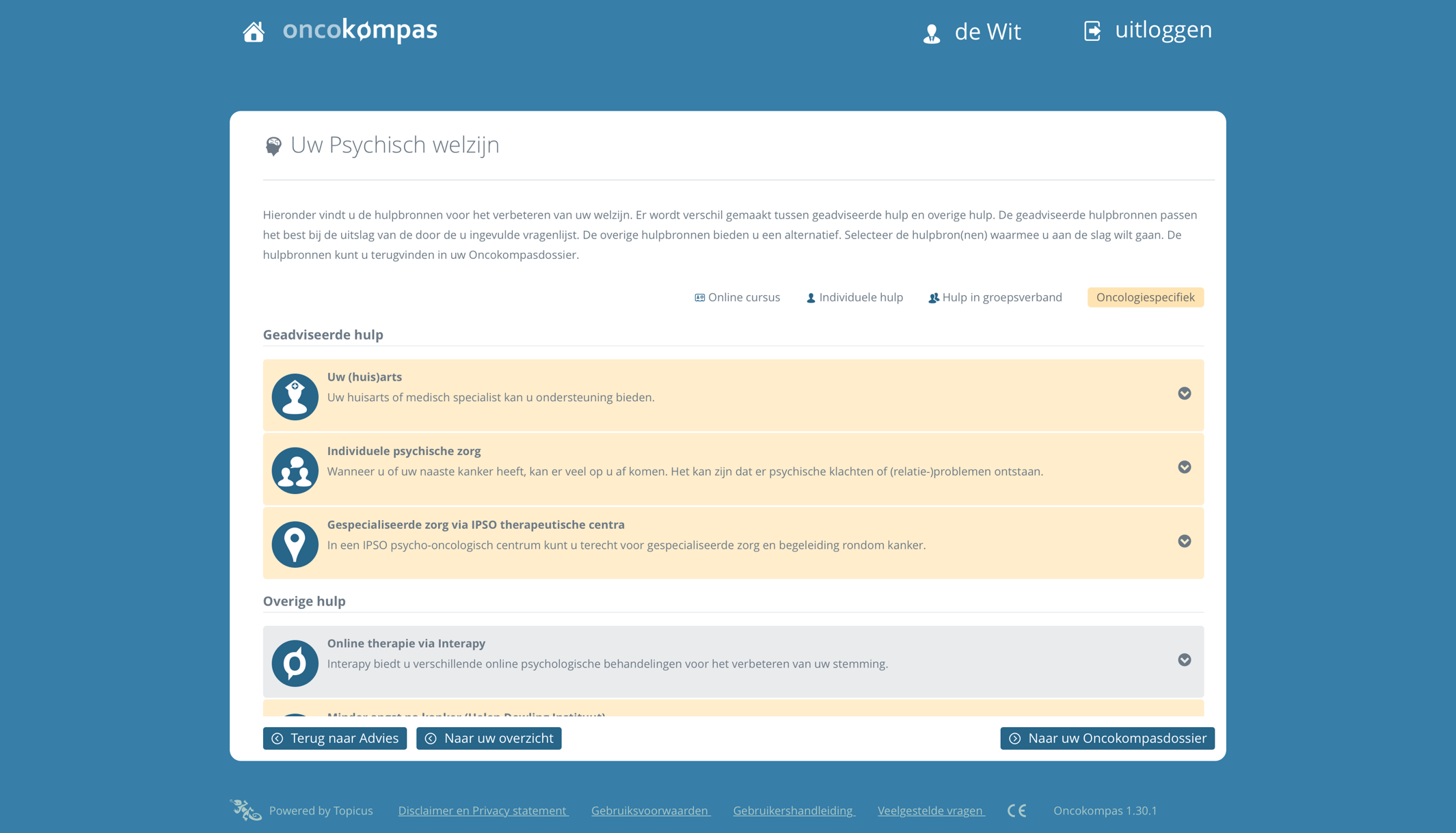


Screenshot 7. Overview of personalized supportive care options in the component ‘Act’.


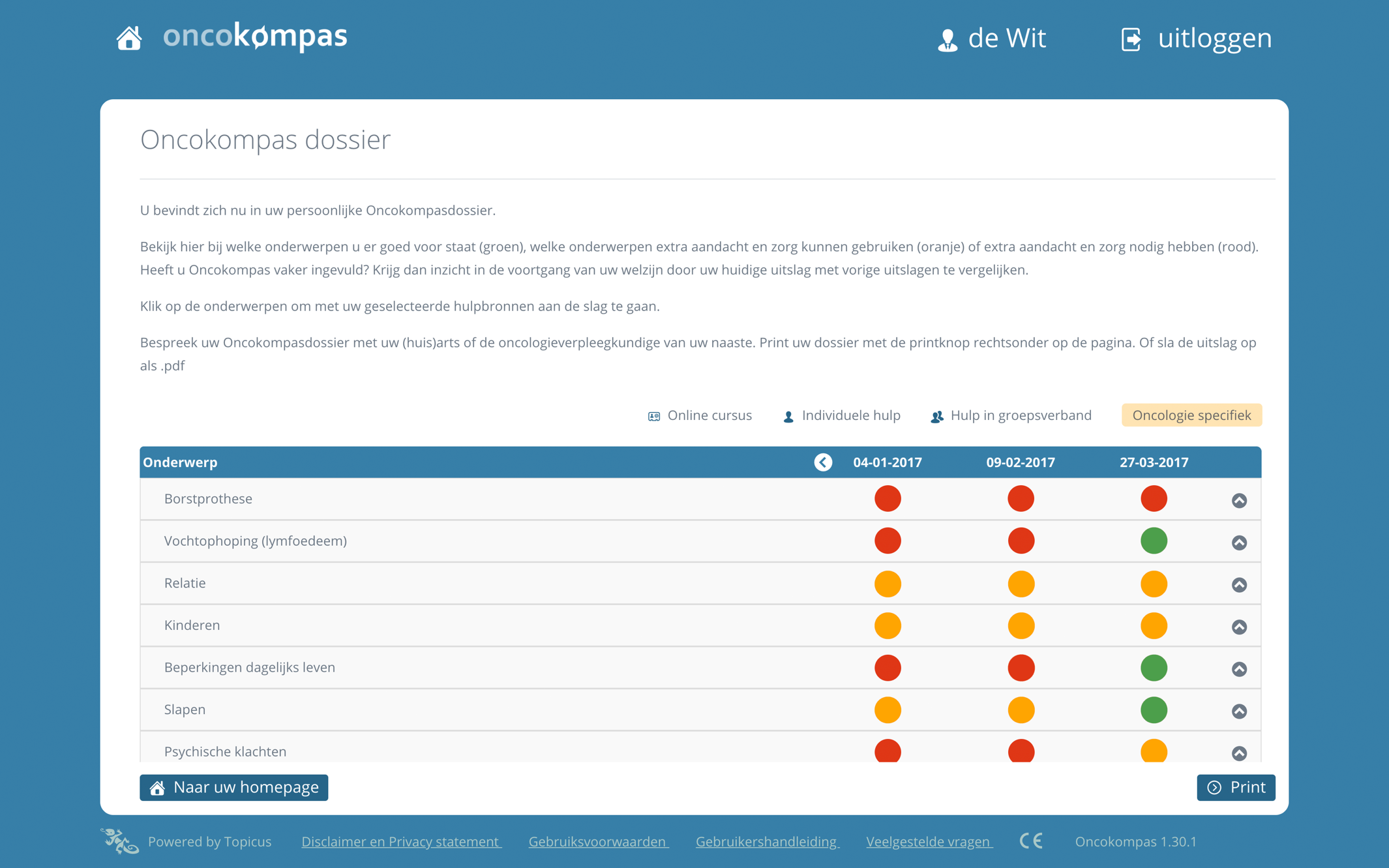


Screenshot 8. Oncokompas dossier function.
