# Supplementary material for: Adoption and implementation of a web-based self-management application “Oncokompas” in routine cancer care: a national pilot study
Source: Support Care Cancer. 2018 Dec 18;27(8):2911–20. doi: 10.1007/s00520-018-4591-5 (PMC6598735; doi:10.1007/s00520-018-4591-5)
Supplement: Supplementary file 2 — (DOCX 31 kb) [file 520_2018_4591_MOESM2_ESM.docx]

**Evaluation survey Oncokompas implementation**

We are evaluating the use of Oncokompas in clinical practice in the context of the current Oncokompas implementation. We are keen to hear your opinions in working with Oncokompas. With your answers, we will be able to improve the implementation.

Filling out the survey will take 10 minutes of your time. Your answers will only be used for evaluation purposes. We thank you for your participation.

1. **What is your position?**

- Medical specialist
- Resident
- Nurse practitioner
- Nurse specialist
- Nurse
- (Physician-) assistant
- Other: ____________________

1. **In what hospital do you work?**
2. **How many new patients with cancer do you see on a yearly basis?**

- 1 to 5 patients
- 6 to 10 patients
- 11 to 50 patients
- More than 50 patients
- None

1. **To what extent are you informed about the content of Oncokompas?**

- I’m not familiar with Oncokompas
- I'm familiar with Oncokompas, but I have not gone through it (yet)
- I'm familiar with Oncokompas and I have gone through the clickable demo
- I'm familiar with Oncokompas and I have gone through it completely

1. **Did you offer Oncokompas to your patients yourself?**

- Yes
- No

1. **To how many of your patients has Oncokompas been offered?**

- 1 to 5 patients
- 6 to 10 patients
- 11 to 50 patients
- More than 50 patients
- None

1. **If you did not offer Oncokompas one time or the other where it was possible though, what was the reason for this? *(multiple answers possible)***

- I don’t have time to register patients for Oncokompas
- This was done by somebody else (e.g. a nurse or physician)
- I forgot to register the patient for Oncokompas
- I don’t endorse the use of Oncokompas
- I don’t endorse the content of Oncokompas
- Other: ____________________

1. **With how many of your patients did Oncokompas come up in a follow up consult?**

- 1 to 5 patients
- 6 to 10 patients
- 11 to 50 patients
- More than 50 patients
- None

1. **How many of your patients brought a print or digital copy of their Oncokompas dossier to a follow up consult?**

- 1 to 5 patients
- 6 to 10 patients
- 11 to 50 patients
- More than 50 patients
- None

1. **Could you please indicate to what extent you agree with the statements below?**

|  | Totally disagree (1) | Disagree (2) | Neither agree nor disagree (3) | Agree (4) | Totally agree (5) |
| --- | --- | --- | --- | --- | --- |
| A. It is clear for me how to invite a patient to Oncokompas. |  |  |  |  |  |
| B. Oncokompas is based on factually correct knowledge. |  |  |  |  |  |
| C. I have all the materials information and materials needed to offer Oncokompas. |  |  |  |  |  |
| D. Offering Oncokompas is too complex for me to use it. |  |  |  |  |  |
| E. Oncokompas is a good match for how I am used to work. |  |  |  |  |  |
| F. The outcomes of usage Oncokompas by patients are clearly observable. |  |  |  |  |  |
| G. I think Oncokompas is relevant for my patients. |  |  |  |  |  |
| H. I think the use of Oncokompas is too burdensome for patients. |  |  |  |  |  |

1. **To what extent does Oncokompas provide you with the following personal benefits?**

|  | Totally disagree (1) | Disagree (2) | Neither agree nor disagree (3) | Agree (4) | Totally agree (5) |
| --- | --- | --- | --- | --- | --- |
| A. Using Oncokompas means I spend less time per patient. |  |  |  |  |  |
| B. Using Oncokompas means I am more easily able to talk with patients about their quality of life and/or symptoms. |  |  |  |  |  |
| C. Using Oncokompas means my patients are more satisfied with the provision of information. |  |  |  |  |  |
| D. Using Oncokompas means I am more in compliance with clinical guidelines and procedures. |  |  |  |  |  |
| E. Using Oncokompas means I refer patients less often to paramedic or psychosocial care. |  |  |  |  |  |
| F. Using Oncokompas results in less hospital expenses |  |  |  |  |  |

1. **To what extent does Oncokompas provide you with the following personal drawbacks?**

|  | Totally disagree (1) | Disagree (2) | Neither agree nor disagree (3) | Agree (4) | Totally agree (5) |
| --- | --- | --- | --- | --- | --- |
| A. Using Oncokompas means I spend more time per patient. |  |  |  |  |  |
| B. Using Oncokompas means patients receive too much information. |  |  |  |  |  |
| C. Using Oncokompas means my hospital spends more on paramedic or psychosocial care. |  |  |  |  |  |

1. **I think it is important to achieve the following objectives with Oncokompas:**

|  | Totally disagree (1) | Disagree (2) | Neither agree nor disagree (3) | Agree (4) | Totally agree (5) |
| --- | --- | --- | --- | --- | --- |
| A. Provide my patients with more insights into their quality of life and/or symptoms, and its course over time |  |  |  |  |  |
| B. Inform my patients about the coherence between symptoms. |  |  |  |  |  |
| C. Provide my patients with good advice on problem areas. |  |  |  |  |  |
| D. Offer my patients appropriate resources. |  |  |  |  |  |
| E. Empower my patients to independently work on their recovery. |  |  |  |  |  |

1. **I expect that using Oncokompas will actually achieve the follow objectives for my patients:**

|  | Most definitely not (1) | Definitely not (2) | Perhaps not, perhaps (3) | Definitely (4) | Most definitely (5) |
| --- | --- | --- | --- | --- | --- |
| A. Provide my patients with more insights into their quality of life and/or symptoms, and its course over time |  |  |  |  |  |
| B. Inform my patients about the coherence between symptoms. |  |  |  |  |  |
| C. Provide my patients with good advice on problem areas. |  |  |  |  |  |
| D. Offer my patients appropriate resources. |  |  |  |  |  |
| E. Empower my patients to independently work on their recovery. |  |  |  |  |  |

1. **I feel it is my responsibility as a professional to:**

|  | Totally disagree (1) | Disagree (2) | Neither agree nor disagree (3) | Agree (4) | Totally agree (5) |
| --- | --- | --- | --- | --- | --- |
| A. Provide my patients with more insights into their quality of life and/or symptoms, and its course over time |  |  |  |  |  |
| B. Inform my patients about the coherence between symptoms. |  |  |  |  |  |
| C. Provide my patients with good advice on problem areas. |  |  |  |  |  |
| D. Offer my patients appropriate resources. |  |  |  |  |  |
| E. Empower my patients to independently work on their recovery. |  |  |  |  |  |

1. **When Oncokompas will be integrated after this pilot implementation project…**

|  | Totally disagree (1) | Disagree (2) | Neither agree nor disagree (3) | Agree (4) | Totally agree (5) |
| --- | --- | --- | --- | --- | --- |
| … then I expect patients in general will actually use Oncokompas |  |  |  |  |  |
| … then I expect patients will generally be satisfied with Oncokompas |  |  |  |  |  |

1. **I can count on adequate assistance from …. In offering Oncokompas.**

|  | Totally disagree (1) | Disagree (2) | Neither agree nor disagree (3) | Agree (4) | Totally agree (5) |
| --- | --- | --- | --- | --- | --- |
| A. Management |  |  |  |  |  |
| B. My colleagues |  |  |  |  |  |
| C. The Oncokompas implementation team |  |  |  |  |  |
| D. The Oncokompas help desk |  |  |  |  |  |

1. **Your colleagues can also offer Oncokompas to their patients. In your opinion, what proportion of the colleagues actually offer Oncokompas to their patients?**

- Not a single colleague
- Almost no colleagues
- A minority
- Half
- A majority
- Almost all colleagues
- All colleagues.

1. **To what extent does …. expect you to offer Oncokompas?**

|  | Most definitely not (1) | Definitely not (2) | Perhaps not, perhaps (3) | Definitely (4) | Most definitely (5) |
| --- | --- | --- | --- | --- | --- |
| A. Management |  |  |  |  |  |
| B. My colleagues |  |  |  |  |  |
| C. The Oncokompas implementation team |  |  |  |  |  |
| D. The Oncokompas help desk |  |  |  |  |  |

1. **When it comes to working with Oncokompas, to what extent do you comply with the opinions of the follow people?**

|  | Very little (1) | Little (2) | Not a little, not a lot (3) | A lot (4) | A great deal (5) |
| --- | --- | --- | --- | --- | --- |
| A. Management |  |  |  |  |  |
| B. My colleagues |  |  |  |  |  |
| C. The Oncokompas implementation team |  |  |  |  |  |
| D. The Oncokompas help desk |  |  |  |  |  |

1. **Are you able to fulfil the actions listed below, when offering Oncokompas?**

|  | Most definitely not (1) | Definitely not (2) | Perhaps not, perhaps (3) | Definitely (4) | Most definitely (5) |
| --- | --- | --- | --- | --- | --- |
| A. Offer Oncokompas to every eligible patient. |  |  |  |  |  |
| B. Explain the use of Oncokompas, if a patient needs that. |  |  |  |  |  |
| C. Ask every patient that used Oncokompas for their experiences using Oncokompas. |  |  |  |  |  |
| D. Discuss Oncokompas with a patient if a patient brings it up. |  |  |  |  |  |
| E. Discuss a print of the Oncokompas dossier with a patient if a patient brings this to a follow up consult |  |  |  |  |  |

1. **I know enough to offer Oncokompas**

- Totally disagree
- Disagree
- Neither agree nor disagree
- Agree
- Totally agree

1. **Has the management set up formal arrangements in your organisation relating to the use of this innovation (in policy plans, work plans and so on)?**

- No
- Yes

1. **In my organisation, one or more people have been designated to coordinate the process of implementing the innovation.**

- No
- Yes

1. **Are there, in addition to the implementation of Oncokompas, any other changes in the organisation affecting the implementation of the innovation now or in the foreseeable future (reorganisation, merger, cuts, staffing changes, other innovations)?**

- No
- Yes

1. **Could you please indicate to what extent you agree with the statements below?**

|  | Totally disagree (1) | Disagree (2) | Neither agree nor disagree (3) | Agree (4) | Totally agree (5) |
| --- | --- | --- | --- | --- | --- |
| A. In my organisation, there are arrangements in place so that staff who offer Oncokompas and leave the organisation are replaced in good time by employees who are/will be adequately prepared to take over. |  |  |  |  |  |
| B. There are enough people in our organisation to use Oncokompas as intended. |  |  |  |  |  |
| C. There are enough financial resources available to use Oncokompas as intended. |  |  |  |  |  |
| D. My hospital provides me with enough time to include Oncokompas as intended in my day-to-day work. |  |  |  |  |  |
| E. My hospital provides me with enough materials and other resources or facilities necessary to offer Oncokompas as intended. |  |  |  |  |  |
| E. When I have questions about Oncokompas, I know where to be. |  |  |  |  |  |
| E. In my organisation, feedback is regularly provided about progress with the implementation of the innovation. |  |  |  |  |  |

1. **The activities listed in Oncokompas fit in well with existing legislation and regulations.**

- Totally disagree
- Disagree
- Neither agree nor disagree
- Agree
- Totally agree

1. **How probable is it that you would recommend your colleagues to offer Oncokompas to their patients?**

(very unlikely) 0 1 2 3 4 5 6 7 8 9 10 (very likely)

1. **How could Oncokompas or the way it is used be further improved?**
